# Multivariate analysis of poverty and social exclusion across EU countries in the context of Sustainable Development Goal 1

**DOI:** 10.1371/journal.pone.0343050

**Published:** 2026-09-30

**Authors:** Silvia Megyesiova, Cecilia Olexova

**Affiliations:** Faculty of Business Economics with seat in Košice, Bratislava University of Economics and Business, Košice, Slovak Republic; University of Coimbra: Universidade de Coimbra, PORTUGAL

## Abstract

Poverty reduction remains one of the socio-economic concerns of the international agenda. This research aims to examine the multidimensional nature of poverty across EU Member States through a multivariate analysis of selected socio-economic indicators. Ten indicators were initially selected to capture the multidimensional nature of poverty and the broader socio-economic situation encompassing income poverty and deprivation within the sustainable development goal 1, housing conditions, economic performance and digital factors. Based on the Kaiser-Meyer-Olkin test and subsequent statistical analyses, six indicators were retained for the final multivariate analysis. Data were drawn from the Eurostat Database. Univariate and multivariate statistical techniques were applied during the analyses. Pearson’s correlation coefficient was used to find linear relationships between pairs of indicators, while a principal component analysis and a cluster analysis were implemented to examine multidimensional structures and patterns. The multivariate analysis was conducted across three time spans: 2015–2019, 2020 (analysed separately as the Covid-19 pandemic disrupted economies) and 2021–2023. The results reveal a general decline in poverty; nevertheless, they also emphasise persistent regional disparities across EU Member States. Bulgaria, Greece, Romania, and Latvia form the most vulnerable cluster in the final period, whereas several countries in Northern and Western Europe show more stable trends. The results further indicate that multidimensional poverty profiles cannot be explained solely by economic performance or geographical location. In conclusion, the cluster-based approach provides a dynamic perspective on the diverse experiences of poverty in the EU. The results show the need for tailored policy responses that reflect the specificities of individual EU countries and clusters to promote sustainable and inclusive development and to strengthen resilience to potential future crises.

## 1 Introduction

Global poverty remains among the most pressing challenges of the present day. According to the latest estimates, as many as 1.3 billion people out of nearly 6 billion individuals covered in 109 countries worldwide were living in multidimensional poverty in 2021, with almost 85% of them situated in sub-Saharan Africa and South Asia, and half being under the age of 18 [1]. In-work poverty also remains a significant global problem. According to the ILO *World Employment and Social Outlook: Trends 2025 in Figures* report [2], approximately 240 million people worldwide belong to the category of extreme working poor, representing approximately 7% of the global labour force. These are employed individuals whose incomes are insufficient to ensure decent living conditions. These findings suggest that assessment of only the unemployment rate is inadequate – the quality and dignity of work are equally crucial. In addition, the ILO report points to persistent disparities in employment between women and men, and the long-standing problem of youth unemployment, which are factors significantly influencing the risk of poverty and structural vulnerability.

Although the European Union (EU) as a whole is in a more favourable socio-economic position, significant disparities between Member States and regions persist despite the EU’s long-standing efforts to reduce poverty and social exclusion. While some EU countries have made progress in achieving Sustainable Development Goal No. 1 (SDG 1) – No Poverty (hereinafter SDG 1), the pandemic crisis, geopolitical tensions, new technological and political risks, and the consequences of climate change have weakened these efforts and, at the same time, exposed systemic inequalities in the field of social protection [3]. Relative poverty in Europe deepened considerably as a result of the Covid-19 pandemic, and in 2020, 96.5M people in the EU27 were at risk of poverty or social exclusion, representing 21.9% of the population [1]. Poverty in the EU is taking on an increasingly complex character. It is no longer solely a matter of income deprivation, but also of material deprivation, availability of infrastructure, or limited access to dwelling [4,5], employment [6,7], digital infrastructure and information literacy [8] and skills, as well as growing environmental threats [9] and changes in household and employment structures with differing impacts on poverty [10].

For this reason, multidimensional approaches to measuring poverty are gaining increasing importance [11–18]. These approaches make it possible to capture the interconnections between material deprivation, employment, digital access, and competencies essential for sustainability. Current research confirms that countries and regions with low levels of digital and environmental skills also display higher levels of vulnerability to poverty [8,12]. Nevertheless, there are only a few comprehensive analyses that integrate multiple dimensions of poverty (including housing, employment, and digitalisation) into a single analytical framework while simultaneously examining them within the European regional context. One way to gain a better understanding of various forms of vulnerability is through the use of multidimensional assessment and the typology of countries based on combinations of multiple factors. Grouping countries according to multiple indicators can reveal common patterns of risk and readiness for current challenges, such as housing costs, digital accessibility, or the level of key economic indicators.

This study responds to the above gap by applying a multivariate analysis of poverty within the EU Member States and by building a three-period cluster typology based on socio-economic indicators covering the following conceptual dimensions: (1) poverty and deprivation – with its core indicators: monetary poverty, inability to afford basic needs, very limited work intensity, and employment below the poverty threshold; (2) housing – often a hidden and overlooked indicator of poverty, yet a fundamental factor on the expenditure side: housing cost overburden and housing deprivation; and (3) the economic and digital context, which reflects the structural readiness of countries to address poverty in the future through the indicators: GDP in PPS, GDP growth, high-speed internet coverage, and digital skills. The objective is to determine homogeneous groups of EU countries in order to assess how successfully, or unsuccessfully, they are progressing towards achieving SDG 1 in all its dimensions at the global level.

We address the following research questions:

- How do EU countries group into internally congruent clusters when measured by a common set of indicators covering poverty/deprivation, housing, and economic/digital context?
- How stable or mobile are these clusters across three time spans (2015–2019; 2020; 2021–2023), and what does this reveal about persistent vs. transitional disadvantage (including the pandemic shock)?

Using cluster analysis, we identify country profiles representing different types of poverty risk and levels of readiness, or lack thereof, to meet the challenges of the twin transition, the parallel shift towards a digital and sustainable economy across the EU, with the resulting clusters reflecting different types of socio-economic situations in line with SDG 1.

These findings may support more precisely targeted public policies and to the promotion of inclusive social development in accordance with the 2030 Agenda and the European Pillar of Social Rights, in order to lower poverty in all its forms and dimensions.

## 2 Research background

### 2.1 Dimensions of poverty and social vulnerability in the European context

Poverty is recognised as a multidimensional phenomenon that cannot be adequately captured by income measures alone. While monetary indicators remain important for assessing economic disadvantage, they do not fully reflect differences in living conditions, access to housing, health, education, employment opportunities, or the ability to participate in society. Alkire and Apablaza [19] showed that poverty in Europe is shaped not only by income and material deprivation but also by a range of non-monetary dimensions, highlighting the importance of considering multiple dimensions simultaneously. The literature review focuses on three thematic areas relevant to the multidimensional understanding of poverty: income and material deprivation, housing conditions, and the broader economic-digital context.

#### 2.1.1 Poverty and deprivation in the European context.

Poverty is a complex and multidimensional phenomenon that goes beyond low income and also includes limited opportunities to access basic needs, services, and opportunities. Within the EU, the AROPE indicator has long been used to monitor poverty, which reflects exposure to poverty and social exclusion. This approach reflects the recognition that poverty is not solely financial but affects multiple areas of an individual’s and household’s life [20].

Nevertheless, some authors point to methodological shortcomings of this indicator, particularly its “at risk of poverty” (AROP) component, which in fact captures relative income disparities rather than absolute poverty, as it is based on median income [21]. These limitations complicate the interpretation of results and reduce comparability between EU countries, especially for those with differing income structures and welfare systems. Despite some limitations, the AROP indicator remains highly relevant in the context of developed economies, where poverty is understood as a relative phenomenon associated with the ability to participate fully in the standard of living considered acceptable within a given society. In this context, income poverty reflects not only a lack of financial resources but also the risk of social exclusion. Alkire and Apablaza [19] likewise argue that income-based indicators should not be abandoned but complemented by additional dimensions such as employment, material deprivation, education, health and living conditions in order to capture the multidimensional nature of poverty more comprehensively.

A complementary perspective is offered in the paper of Jankiewicz [22], which suggests that a shift in the economic structure towards services can significantly improve social sustainability, as defined through the goals SDG 1–3.

In the context of the CEE region´s transition economies, there is therefore a growing need to identify structural factors influencing socio-economic inequalities in the region and vulnerability to poverty. A significant contribution in this area is provided by the study of [16], in which the authors, using panel data analysis, examined the role of the labour market, globalisation, economic development, and the quality of governance. The results indicate that improvements in these areas can substantially contribute to reducing inequalities. Particular emphasis is placed on the effect of minimum wages – an increase in the minimum wage can reduce income disparities, especially in emerging countries. The findings also highlight the mixed effects of employment protection legislation (EPL) and emphasise the importance of balanced policies that promote social justice. These insights are crucial for developing effective policy instruments in conformity with the objectives of SDG 1 and SDG 10 (Reduced Inequalities), particularly in the CEE region, where income and regional disparities remain significant.

The COVID-19 pandemic had a markedly negative impact on the implementation of the SDGs in Europe, with the most affected areas being those related to poverty, hunger, and health. As shown by the study of [3], the implementation of SDG 1, SDG 2, and SDG 3 was significantly weakened in CEE countries, revealing systemic deficiencies in social protection and deepening existing social inequalities. Based on comparative analysis, the author concludes that the consequences of the pandemic lay not only in the disruption of economic activity but, above all, in exposing profound structural inequalities that threaten the achievement of the 2030 Agenda. These findings point to the need for more resilient social systems capable of responding more effectively to crisis situations and minimising the impact on at-risk population groups.

In the context of European social policy, in-work poverty is one of the main indicators of social inequality and the failure of traditional mechanisms of social mobility. This phenomenon results from a combination of several factors: low wages, insufficient working hours (often part-time contracts), job instability (e.g., temporary contracts or casual work), inadequate social security, and wage differentials between regions or sectors of the economy [6,7]. As a consequence, there is a growing need to reassess the role of minimum wages, tax and contribution incentives, and employment policy in combating poverty among the employed population.

At the same time, there is a need to analyse poverty in a territorial context as well [23]. A regional analysis of poverty at the NUTS 2 level was carried out by Weziak-Bialowolska and Dijkstra [24], with the results confirming significant differences in poverty rates between individual regions within EU countries. The results also showed that regions including capital cities display lower poverty rates than the national average (e.g., Bratislava, Bucharest, Budapest, Prague, Sofia), with the exception of Vienna, Brussels, and Berlin, where poverty was higher than the national average.

Similarly, an analysis of the regional performance of Bulgaria and Romania in the areas of poverty, education, and decent work pointed to significant disparities between macro-regions and identified specific problems in rural areas, including informal work and low participation in social and health insurance systems, which contribute to social vulnerability and distort official unemployment statistics [25].

#### 2.1.2 Housing as a dimension of poverty.

In recent years, traditional income-based dimensions of poverty have increasingly been complemented by indicators related to housing deprivation (e.g., inadequate housing conditions, poor sanitary conditions, or overcrowding), which also reflect the impact of housing type and location. Illustratively, analysis of data for Poland distinguishes three dimensions of housing deprivation (overcrowding only, housing-quality problems only, and severe housing deprivation). The results show that the effects of socio-economic characteristics differ across these dimensions; holding other covariates constant, housing-quality problems are more pronounced in rural areas, whereas the likelihood of overcrowding and severe housing deprivation is higher in urban areas [5]. These phenomena are closely linked to material deprivation and relative poverty, thereby significantly broadening the analytical framework of poverty in the European context [26]. As Dewilde [4] points out, housing is becoming a growing source of social inequalities, particularly in urban areas with high housing costs and insufficient availability of public housing. For this reason, developments in the real estate market are increasingly the subject of public and political debate [27].

Declining conditions of life for households with limited financial resources in some Western European countries demonstrate that poverty analysis needs to complement the income perspective with the expenditure side, with a particular focus on housing costs.

The housing cost overburden rate indicates households spending more than 40% of their net disposable income on housing [28]. This indicator captures the economic burden on households, that is, situations where high housing expenditures limit their capacity to cover other everyday essentials. Redistributive policies such as rent regulation and accommodation allowances have proven to be instruments capable of mitigating the adverse effect of low income on life quality, while promoting social inclusion throughout the wider society. Conversely, rising property values and their fluctuations correspond to an increased residential deprivation among both tenants and financially vulnerable homeowners [4]. The present results confirm that poverty reduction policies should systematically take housing into account as a basic need, within the broader context of living costs and the accessibility of public services.

#### 2.1.3 Economic and digital context of social vulnerability.

With the rise of digitalisation in both the economy and public services, digital inclusion is emerging as a necessary precondition for social inclusion. Deficient access to high-speed internet, along with a lack of digital skills, constitute risk factors that can reduce individuals’ employability and thereby exacerbate poverty [29]. Digital exclusion is often interconnected with other forms of disadvantage, such as low educational attainment, rural location, or low income.

Current EU economic and political strategies are based on a double transition – not only towards a digital but also towards a sustainable economy – referred to as the twin transition. This process requires fundamental alternations in the configuration of the labour market and in workers´ competencies. In this context, Decision (EU) 2022/2481 outlines ambitious digital targets for 2030, that include widespread basic digital competences and comprehensive access to fast networks [30]. These targets constitute a strategic framework aimed at reducing digital inequality and increasing resilience to social and regional disparities.

The importance of regional differences is also indicated by research findings showing that, after including regional specificities in analyses through fixed effects, variables such as education or the employment rate do not display a significant impact. In contrast, the local GDP per capita demonstrates a consistent and robust relationship with the living standards of the least affluent groups. The analysis emphasises the relevance of a spatial dimension in the design of inclusive policies [23].

The economic performance of countries is vital for the formation of their capacity to respond to social challenges, including poverty and inequality. GDP in PPS is a relevant indicator of overall living standards and the stage of economic progress, while the rate of GDP growth can signal a country’s ability to create new jobs, invest in public services, and manage structural changes. Higher economic performance is often associated with better infrastructure, higher-quality services, and lower levels of material deprivation, particularly when combined with effective redistributive policies. Conversely, low or stagnant economic growth can reduce the state’s capacity to address poverty in a systemic and long-term manner. Empirical studies confirm this relationship – a 10% increase in GDP leads to a reduction in multidimensional poverty of approximately 4–5% [13]. Similarly, GDP per capita was identified as a significant factor in reducing the risk of poverty within the EU [31]. Nevertheless, the relation between GDP and income inequality is non-linear; in accordance with the Kuznets hypothesis, developing economies may experience a temporary increase in inequalities unless offset by social policies. In later stages of development, such inequality stabilises or declines. Nevertheless, economic growth alone, without reducing income inequality, might not be enough to achieve the EU’s poverty reduction goals [21].

Socio-economic differences between countries and regions are also closely linked to the level of digital and sustainable skills, which several studies identify as significant, since countries and regions with low levels of digital and green skills simultaneously exhibit higher poverty rates and lower resilience to social and/or economic shocks. Qi et al. [8] illustrate this with the example of farming households in China, where they found that improvement of digital skills significantly reduces households’ vulnerability to poverty, thereby mitigating the risk of falling back into poverty. The heterogeneity analysis revealed that this effect was most pronounced in the group of low-income households. Urbanisation without adequate green infrastructure and energy-efficient solutions leads to increased environmental burdens and deepening regional inequalities, which has a direct link to social fragility [32]. Similarly, low media and scientific literacy increase the population’s vulnerability to disinformation, which can undermine trust in institutions and reduce society’s capacity to respond to risks related to elementary needs, including food security, thereby indirectly contributing to the deepening of poverty (SDG 1) [33].

In this context, the development of financial inclusion, supported by digital technologies and financial literacy, can significantly reduce social vulnerability by improving access to credit, supporting entrepreneurship, and strengthening households’ capacity to respond to economic shocks, particularly in rural and low-income regions [34].

Interventions by the European Social Fund (ESF) are instrumental in lowering poverty levels (SDG 1) in less developed regions of the EU, particularly through vocational education, job creation, and infrastructure development [35]. This impact is stronger in less affluent countries, where the ESF helps to address structural barriers in the labour market.

An essential input to linking the social and environmental aspects of resilience is offered by the study of Zeb et al. [9], which analysed the effects of poverty (POV), inequality (GINI), and green economic growth (GEG) on environmental sustainability, measured by the ecological footprint (ECF), across the Belt and Road Initiative (BRI) economies. Their results indicate that higher levels of poverty increase environmental burden throughout the entire distribution, whereas inequality affects environmental quality negatively, particularly in the middle and upper quantiles of the distribution. Green growth shows mixed effects. These findings confirm that environmental sustainability cannot be reached without concurrently addressing poverty and inequality, and that policymaking should reflect the specific characteristics of individual regions. The results are also relevant for EU countries, as they emphasize the need for an integrated and inclusive climate policy that is sensitive to social disparities and vulnerability risks. Energy poverty weakens progress towards environmental goals and negatively affects people’s perceived quality of life across all income groups, with lower middle-income countries being almost three times more affected than high income ones [36]. The long-term social consequences of energy poverty are particularly visible in lower middle-income countries. Therefore, sustainable policy frameworks should address both tangible and intangible dimensions of deprivation simultaneously [37]. Recent studies [38] emphasise that energy poverty represents multidimensional form of deprivation that is linked not only to income levels but also to energy transition and social vulnerability. This approach extends the traditional understanding of poverty by incorporating new dimensions related to access to energy infrastructure availability, and the capacity of households to cope with rising energy costs, all of which are integral to achieving green growth, climate neutrality, and SDG13.

Vulnerability to poverty is increasingly perceived as a complex condition arising from a combination of structural risks – low income, poor access to housing, unstable employment, or poor health status. Persistent inequalities in education, health, and food security increase the likelihood of long-term poverty and weaken the resilience of individuals and households to shocks [12]. These findings confirm the need for a multidimensional understanding of poverty, integrating income, expenditure, regional, and social perspectives. They also underscore the importance of public policies that specifically address the vulnerabilities of different population groups and ensure their inclusion in the fundamental socio-economic fabric of society.

Insufficient competencies among the population can restrict their capacity to adapt to new job market demands and to engage in new forms of economic activity, thereby increasing the risk of persistent unemployment or deepening lack. For this reason, competences should be understood as a crucial mechanism of resilience to poverty – not only as an individual employability factor but as a systemic component of an inclusive and fair transition towards a digital and climate-neutral society.

### 2.2 Multidimensional approaches to assessing poverty and sustainable development

Multidimensional poverty in the EU is taking on an increasingly complex form, requiring new analytical approaches and an emphasis on the combination of social, economic, and spatial factors. Complementing traditional indicators with factors such as housing, digital infrastructure, and competencies enables a better recognizing of the deep structural inequalities which determine the risk to be socially excluded.

Evaluating poverty has progressed beyond traditional methods grounded exclusively on income indicators towards multidimensional frameworks that take into account a broader spectrum of living standards. A significant methodological shift was introduced through the development of a multidimensional poverty measurement framework [11,14]. This approach introduced an innovative identification method and a set of various poverty indices, that allow to capture a more complex reality of poverty through multiple dimensions of deprivation. Owing to its robustness and flexibility, this methodology has become the background for the Multidimensional Poverty Index (MPI) and moderate MPI (MMPI). Nowadays, this index is used in many countries as a primary tool for capturing different forms of vulnerability, including other aspects, e.g., educational factors, health, and living conditions. Approaches combining multiple dimensions of poverty, may also serve as an inspiration for European social policy, particularly in relation to the inclusion of housing, digital access, and environmental risks, which are not yet part of standard indicators such as AROPE [1].

In this European context, using EU-SILC data, rankings of EU countries differ when material deprivation indicators are considered alongside income measures [39]. The findings suggest that income poverty alone does not fully capture living conditions across Member States and support the inclusion of deprivation-based indicators in multidimensional poverty assessment. Alkire and Apablaza [19] applied the Alkire-Foster methodology to EU-SILC data and demonstrated that poverty is influenced not only by income and material deprivation, but also by employment, education, health, and living-environment conditions. Their findings support the use of multidimensional approaches in European poverty assessment.

To better understand regional differences and country performance in measuring poverty and related SDG targets, cluster analyses are increasingly used. The Ward’s method was applied to group 45 Asian countries according to their performance in achieving the Sustainable Development Goals across all four dimensions – economic, social, environmental, and institutional [40]. The results showed that reaching the SDGs is not strictly determined by geographical location and also confirmed the presence of positive correlations between the social and institutional dimensions, alongside between the environmental and economic dimensions.

The analytical framework was expanded to 110 countries worldwide, using the non-hierarchical K-means method [17]. Its aim was to classify countries according to their progress in achieving the SDGs while also taking into account their social, economic, politic and cultural characteristics. According to the analysis, countries with a more stable and developed socio-economic structure and favourable politico-cultural conditions demonstrate significantly better progress in meeting the Sustainable Development Goals. The empirical findings also pointed to a positive relation between socio-economic and politico-cultural indicators and selected SDG indicators. These insights emphasize the worth of multidimensional assessment not only in measuring poverty but also in the general evaluation of country performance from the 2030 Agenda perspective.

In the European context, cluster analysis (hereinafter CA) has been applied, for example, for assessment of the risks of poverty and social exclusion among persons with disabilities in comparison to those without disabilities in EU Member States [15]. Based on 2018 Eurostat data, the author analysed indicators such as low work intensity, low income, material deprivation, the at-risk-of-poverty rate, severe disability, living conditions, and the complete risk of poverty or exclusion of households. Using Ward’s agglomerative method, she identified two main clusters of countries in each group (with disabilities / without disabilities), with 19 countries in the case of persons with disabilities being classified into the cluster with a higher danger of poverty or societal exclusion.

These approaches emphasize the value of multidimensional poverty assessment and the usefulness of cluster analysis as a tool for identifying regional similarities and differences that can support more targeted and effective public policymaking. Beyond clustering, a positional TOPSIS with bilinear ordering can also rank countries by joint poverty and material deprivation, distinguishing three states: persistent severe poverty, less visible poverty and absence of severe poverty [41].

According to this shift towards multidimensional approaches, a systematic bibliometric review of societal protection programs and their impact on reducing vulnerability to poverty, with a particular focus on SDG 1 was provided [12]. The analysis of 127 studies published between 2005 and 2023 points to the dominance of a one-dimensional perspective on poverty – particularly in relation to food security, climate shocks, and health risks. The findings confirm that fiscal redistribution policies (e.g., direct transfers, social security, nutrition support) have a positive effect on reduction of poverty and inequality. However, the authors emphasise the necessity of more comprehensive policy frameworks that connect social protection with environmental sustainability (e.g., SDG 13), thereby supporting more effective implementation of the 2030 Agenda as a whole [12]. From a capacity standpoint, poverty is not just about income; it is a shortfall in what people can actually do and be. Multidimensional deprivation therefore captures these deficits more faithfully [42].

Furthermore, it is important to consider the temporal dimension of deprivation [43]. Analysing EU-SILC longitudinal data, persistent deprivation provides a different picture of poverty than cross-sectional indicators and highlighted the need to distinguish between temporary and long-term vulnerability.

These insights form the basis for the selection of indicators and analytical methods, which are described in detail as part of the methodological section. In doing so, the paper contributes a multidimensional cluster typology that distinguishes persistent from transitional disadvantage and pinpoints policy-salient gaps in housing, in-work poverty, and digital readiness, thereby complementing existing ranking approaches and guiding targeted interventions.

## 3 Methodology

### 3.1 Data and sample

In 2015, the United Nations Member States adopted the 2030 Agenda for Sustainable Development [34,44]. The Agenda comprises 17 Sustainable Development Goals (SDGs), of which SDG 1 aims to eradicate poverty in all its forms worldwide. To monitor progress towards SDG 1 in the EU countries, Eurostat developed a set of indicators [45]. For the purposes of this study, these indicators were complemented by additional socio-economic variables, including GDP per capita in PPS, annual real GDP growth, and indicators related to the EU digital decade. Given the growing importance of digitalisation for labour market (working platform), education and public services (online education, e-government services aimed at individuals and companies, e-Health projects), indicators of the digital decade transformation were included [46]. In total, ten indicators were used for analytical purposes of the poverty and socio-economic setting in the EU member states:

***x***_**1**_ (persons at risk of monetary poverty after social transfer; %),
***x***_**2**_ (severe material and social deprivation rate; %),
***x***_**3**_ (persons living in households with very low work intensity; %),
***x***_**4**_ (in work at-risk-of-poverty rate; %; share of persons who are employed and have an equivalised disposable income below the risk-of-poverty threshold),
***x***_**5**_ (housing cost overburden rate; %),
***x***_**6**_ (severe housing deprivation rate; %),
***x***_**7**_ (GDP per capita in PPS; PPS),
***x***_**8**_ (annual real GDP growth rate; %),
***x***_**9**_ (high-speed internet coverage; %; digital Decade target by 2030 of indicator *x*_9_ in the EU is set to: 100%),
***x***_**10**_ (share of individuals having at least basic digital skills; %; digital Decade target by 2030 of indicator *x*_10_ in the EU is set to: 80%).

The datasets for the indicators were downloaded from the openly available Eurostat Database which provides both the data and metadata for the EU member states and for some non-member countries [45]. The detailed description of the indicators used can be found in S1 Table in S1 File. The corresponding two-letter country codes used throughout the analysis are listed in S2 Table in S1 File.

The multivariate analysis, namely CA of the analysed countries using ten selected indicators was done in three-time spans. The aim was to discover the groups of countries that are very similar regarding to the indicators of poverty and other socio-economic variables and to follow the possible changes of clusters of these countries in three time spans. The first-time span represents the annualized data of the years 2015–2019. The second period corresponds to the year 2020 when the EU economies were mostly hit by the COVID-19 pandemic. Although pandemic effects continued beyond 2020, the year 2020 was analysed separately due to its shock nature, characterized by widespread lockdowns, strict mobility restrictions, abrupt labour-market dislocations, extraordinary policy interventions, and from a methodological standpoint, discontinuities in the time series. The third period deals with the annualized indicators over the time span 2021–2023. Only in exceptional cases, due to lack of full time series of the statistical dataset of some indicators the periods were defined individually.

The main aim of the study was to offer a detailed analysis of the indicators of the SDG 1 and other socio-economic indicators including variables that are intended to follow the digital decade targets of the EU countries.

### 3.2 Statistical methods

Univariate [47,48] and multivariate statistical analytical approach during the analysis were applied. The linear relationship between a pair of variables was identified using Pearson’s correlation coefficient (*r*_*xy*_). In a multidimensional context, the cluster analysis (hereinafter CA) and the principal component analysis (hereinafter PCA) were conducted. CA is an effective analytical approach that attempts to determine structures within a selected dataset. For classifying objects (in our research, EU member states), into clusters, a range of various algorithms and methods can be employed. These methods did not result in substantial changes between dependent or independent indicators, and they established a way to organize objects into groups or clusters where members of a cluster share specific properties in common [49–51]. CA groups objects sharing similar characteristics (in our research EU countries), where countries are similar within a cluster and differ from the objects in different clusters. It can be said that the degree of association between the countries is maximal if they are in the same cluster and minimal in other cases [52]. Various measures can be employed to assess the dissimilarity between objects depending on the type of data and several hierarchical or non-hierarchical procedures can be chosen to determine which clusters should be merged at each step of the analyses, e.g., nearest neighbour method, centroid method, furthest neighbour method, k-mean clustering method, average linkage method, Ward’s method [53,54]. For clustering, all 27 EU member states served as objects and the indicators selected from the Eurostat database were employed as variables. The *r*_*xy*_ of indicators was examined before the clustering since strong linear correlation is a typical issue for multivariate statistical analysis. The PCA was applied to mitigate the problem of a high linear correlation among the indicators [55,56]. The main aim of the PCA is to decrease the dataset dimensionality by transforming it into a smaller number of uncorrelated components. The most important principal components were utilized in the study to conduct the CA. The first principal component captures the largest share of variability in the analysed dataset and each subsequent component accounts for as much of the leftover variability as possible [55–58]. For the next proposed multivariate analysis, it is reasonable to use only the first “*m”* eigenvectors that account for a specified cumulative threshold value for the total variability. The cumulative proportion of variance explained, used as a criterion for determining the number of components, should reach at least 70% [58,59]. Another way to determine the number of components is by using the Kaiser criterion. Based on this rule, a component should only be retained if its eigenvalue is at least 1; in other words, the component must explain at least as much variance as a single variable observed [59,60]. PCA is an effective statistical technique that reduces the dimensionality of input dataset and generates a new set of uncorrelated components, which can then be applied in further multivariate analysis.

## 4 Empirical results

To monitor changes in the indicators and the progress in achieving the goals of the EU countries in regards of the SDG 1, univariate and multivariate analyses were chosen. The analysis of the variables was further supplemented by correlation analysis.

The level of the share of persons at risk of monetary poverty after social transfers (x_1)_ achieved an EU-27 average level of 17.4% in 2015 and it declined to 16.2% in 2023. The decline of the monetary poverty in the time span from 2015 till 2023 was very slow and the situation was negatively affected by the COVID-19 (see Fig 1). In 2015 the monetary poverty was higher than 22 percent altogether in four EU countries (Spain, Lithuania, Latvia, Romania) while in 2023 it was a reality only in two of the EU countries (Estonia, Latvia). The most significant improvement between 2015 and 2023 was achieved in Romania, where the percentage of persons at risk of monetary poverty declined from 25.4% to 21.1%, it means that it improved by 4.3 percentage points (p.p.). A worsening situation in regarding of the monetary poverty was reached in Luxembourg where the value of *x*_1_ increased by 3.5 p.p. (from 15.3% in 2015 to 18.8% in 2023). The lowest share of the *x*_1_ indicator was reached in Czechia where less than 10% of persons are considered as monetary poor.

**Fig 1:**
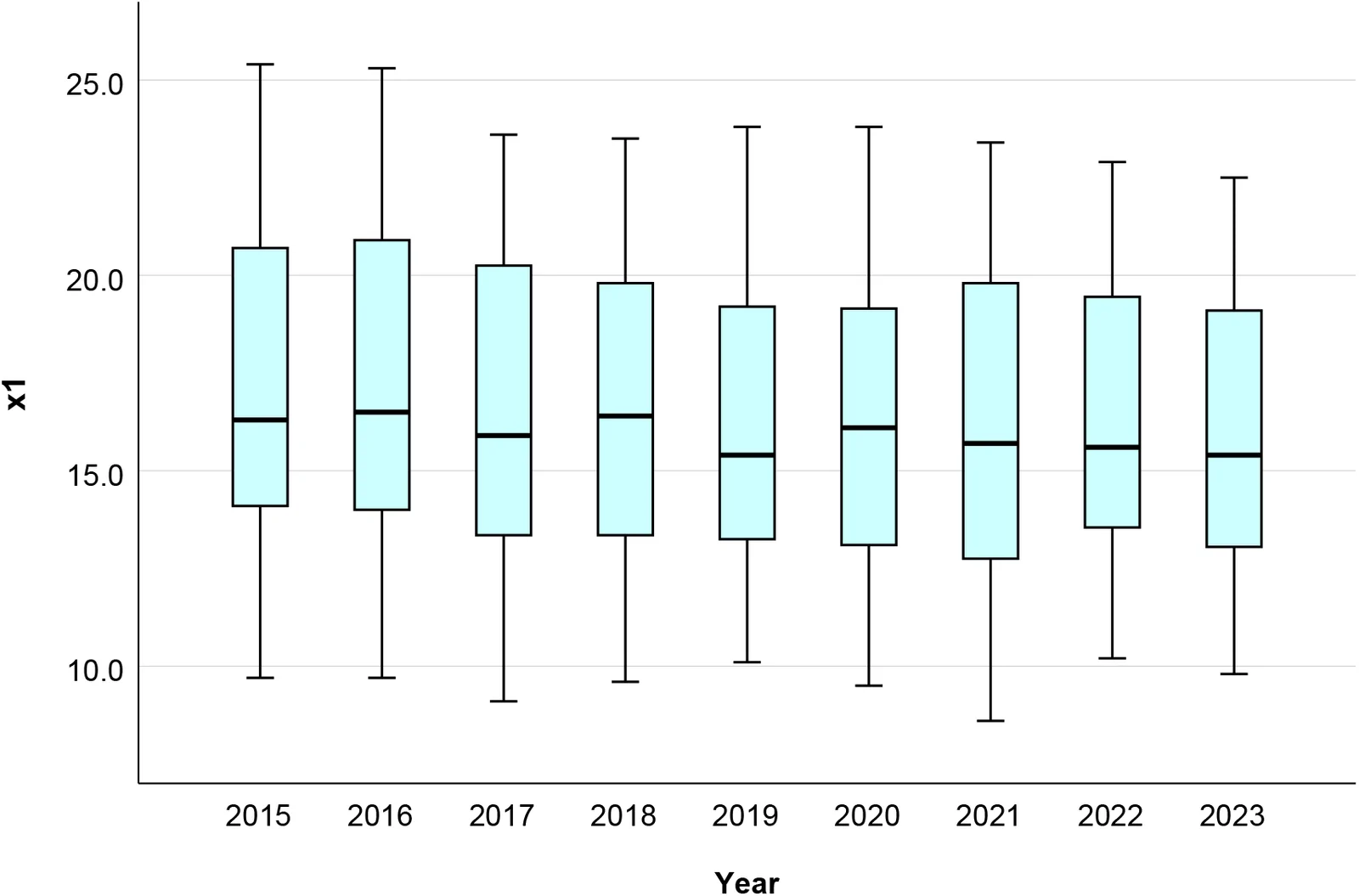
Box plot of the share of persons at risk of monetary poverty after social transfers, %. Source: Eurostat, own presentation of *x*_1_.

The indicator of the share of severely materially or socially deprived persons (*x*_2_) improved significantly from an average EU-27 level of 9.7% in 2015 to only 6.8% in 2023 (Fig 2). Extremely high was the rate of persons severely materially or socially deprived in Bulgaria and Romania, but across both countries, the *x*_2_ indicator fell sharply, a direction that, given the indicator´s scale, corresponds to better performance. In Bulgaria the rate dropped from 36.8% in 2015 to 18.0% in 2023 and in Romania it declined from 34.0% to 19.8% in the same periods. The rate was only about one percent in 2015 in Finland and Sweden while about two percent in Slovenia and Cyprus in 2023.

**Fig 2:**
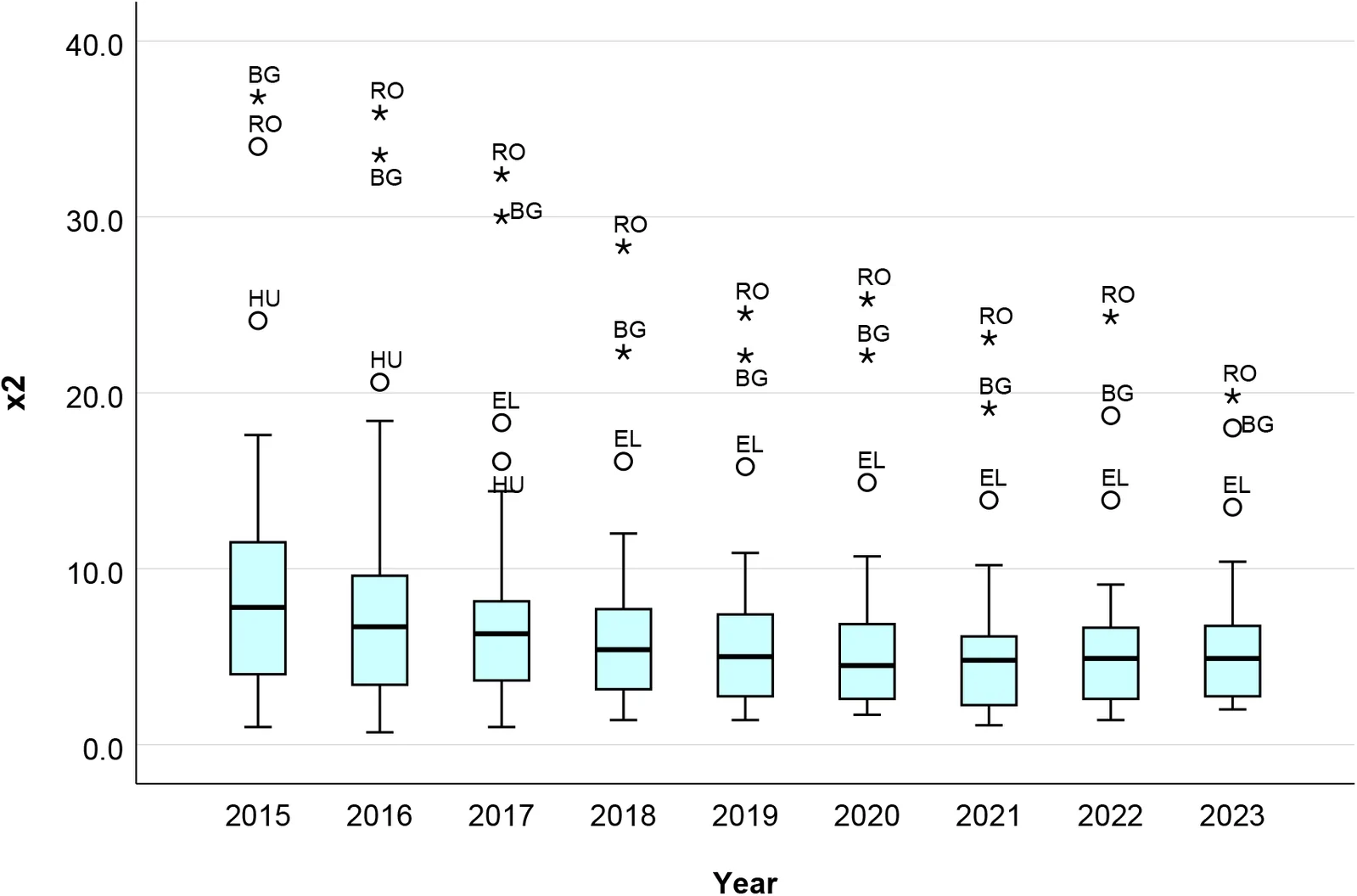
Box plot of the rate of severely materially of socially deprived persons, %. Source: Eurostat, own presentation of *x*_2_.

The value of the share of persons living in household with very low work intensity (x_3_) declined from and EU-27 average level of about 10% in 2015 to 8% in 2023. The indicator declined continuously from 2015 until 2020 when due to the pandemic COVID-19 an increase of the indicator *x*_3_ on EU average level was achieved (see Fig 3). The recovery of EU economies after the COVID pandemic was also reflected in a decline in *x*_3_. Using the box-plot chart only one extreme value of the *x*_3_ indicator was detected, namely in 2015 in Ireland where the percentage of persons living in household with very low work intensity was as high as 18.8%. In 2023 only in Belgium was the rate higher than 10% while in Malta, Slovenia, Luxembourg and Poland it was lower than four percent. Overall, the improvement of the indicator *x*_3_ in the time span from 2015 till 2023 may be represented as progress on this metric.

**Fig 3:**
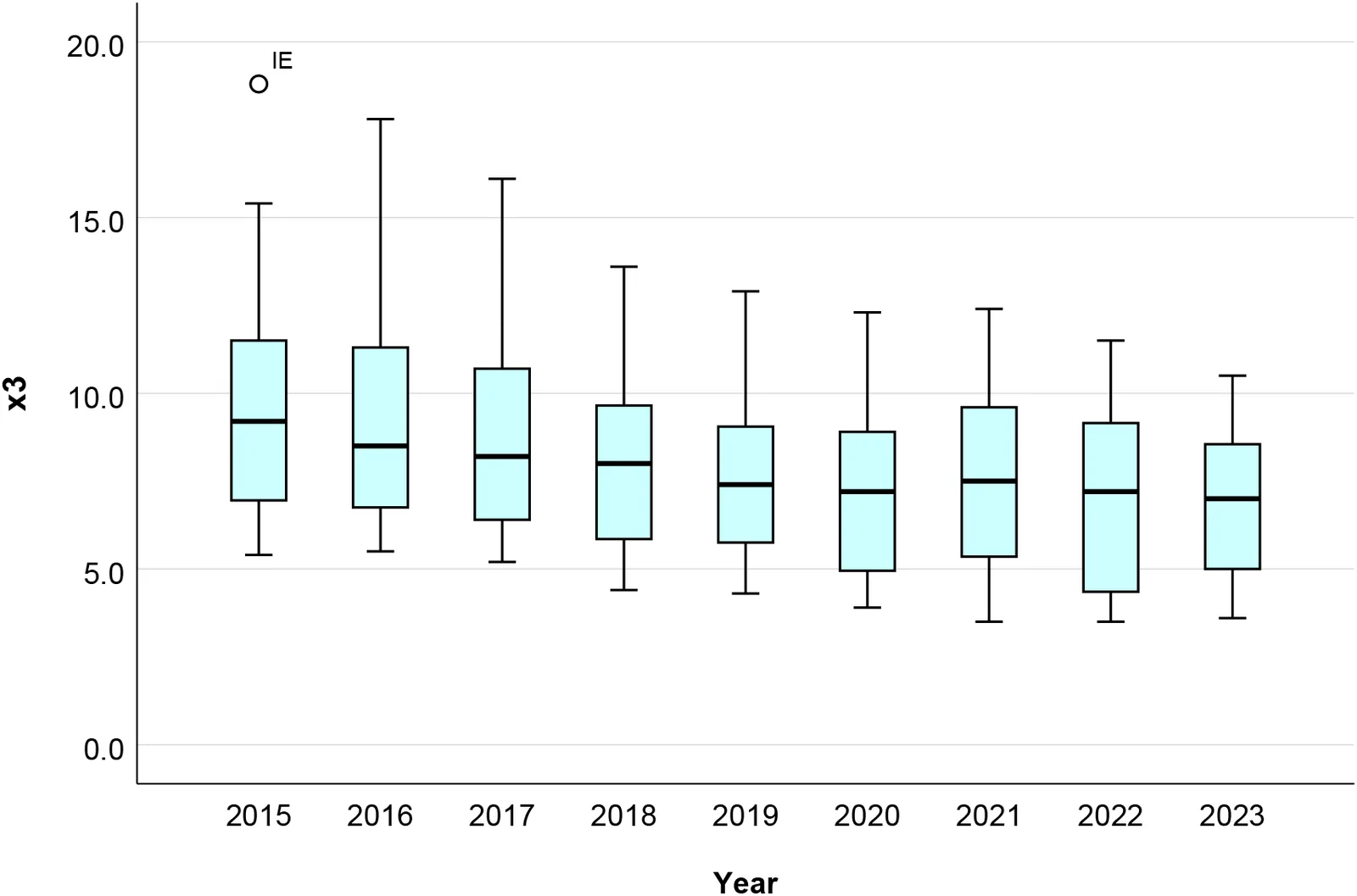
Box plot of the share of persons living in household with very low work intensity, %. Source: Eurostat, own presentation of the indicator *x*_3_.

The share of persons who are employed and have an equivalised disposable income below the risk-of poverty threshold or in-work at risk-of-poverty rate (*x*_4_) changed slightly between 2015 and 2023, the change was for most of the countries positive. In years 2016–2018 also extreme values were detected by the box plot chart (see Fig 4), these extremely high values were achieved in Romania. The three best countries with the lowest share of the in work at-risk-of-poverty were in 2015 Finland (3.5%), Czechia (4.0%), Belgium (4.6%), while to the worst countries belonged Romania (18.8%), Greece (13.4%), Spain (13.1%). At the end of the analysed period the best three countries with the lowest share of the *x*_4_ indicator were again Finland (2.8%), Czechia (3.1%) and Belgium (4.7%). Among the worst countries with the highest share in 2023 again Romania (15.3%) can be found, and also Luxembourg (14.8%) and Bulgaria (11.4%).

**Fig 4:**
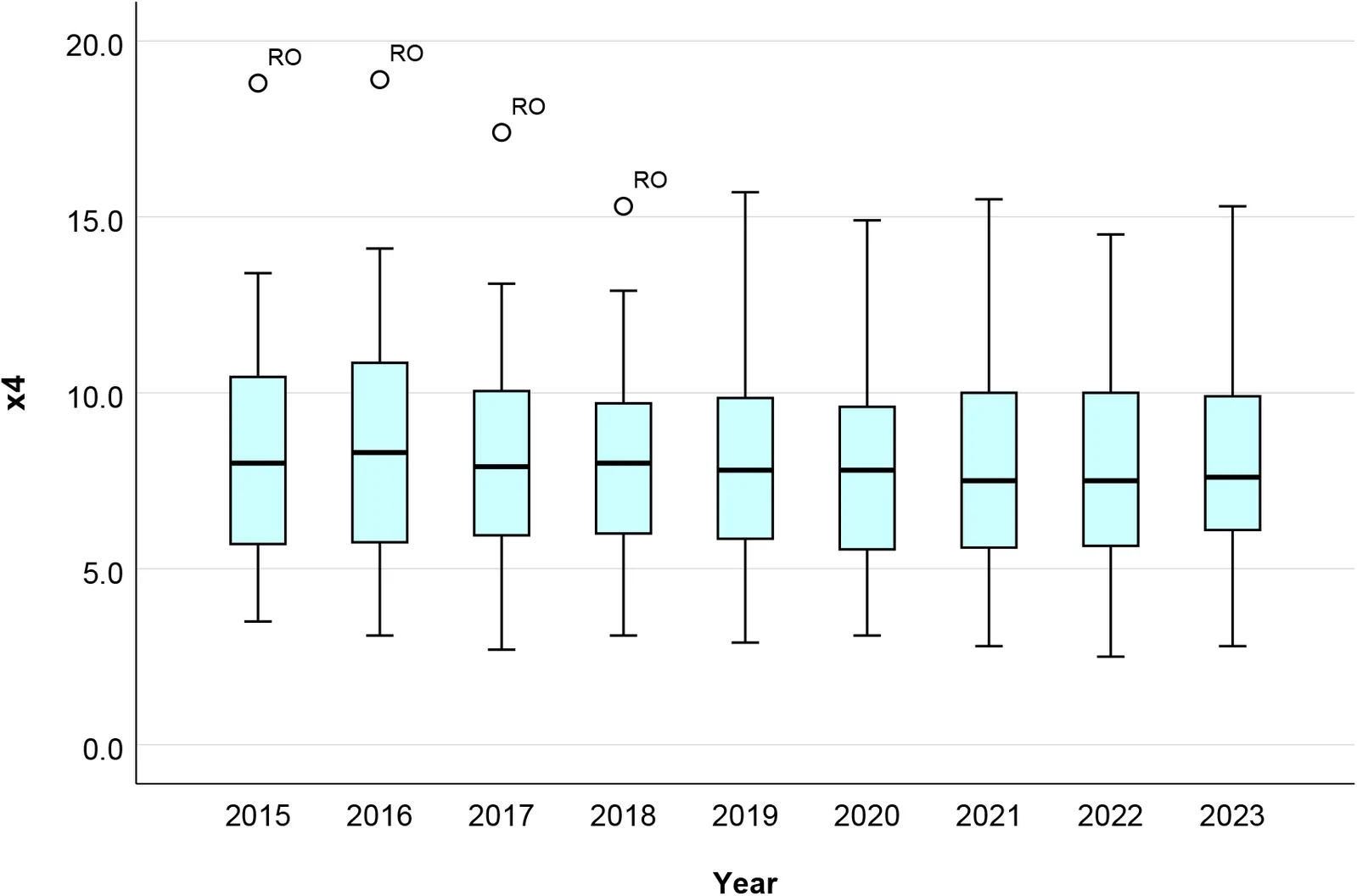
Box plot of the share of persons who are employed and have an equivalised disposable income below the risk-of-poverty threshold, %. Source: Eurostat, own presentation of the indicator *x*_4_.

The housing cost overburden rate (*x*_5_) decreased between 2015 and 2023 significantly especially in Greece, Romania and in the Netherlands. In Greece, where the indicator *x*_5_ was extremely high (see Fig 5), the rate decreased between 2015 and 2023 from 45.5% to 28.5% (by 17 p.p.), in Romania the percentage dropped from 15.9% to 9.1% (by 6.8%). By contrast, over the same span the share increased by 5.5 p.p. in Luxembourg (from 6% to 11.5%) and in Malta by 4.9 p.p. (from 1.1% to 6%), i.e., in the undesired direction for this indicator. In altogether 16 EU countries the percentage of the population living in households that spend 40% or more of the household disposable income on housing was in 2023 lower than eight percent, that is a positive development, since in 2015 only 10 of the EU countries had the share lower than eight percent.

**Fig 5:**
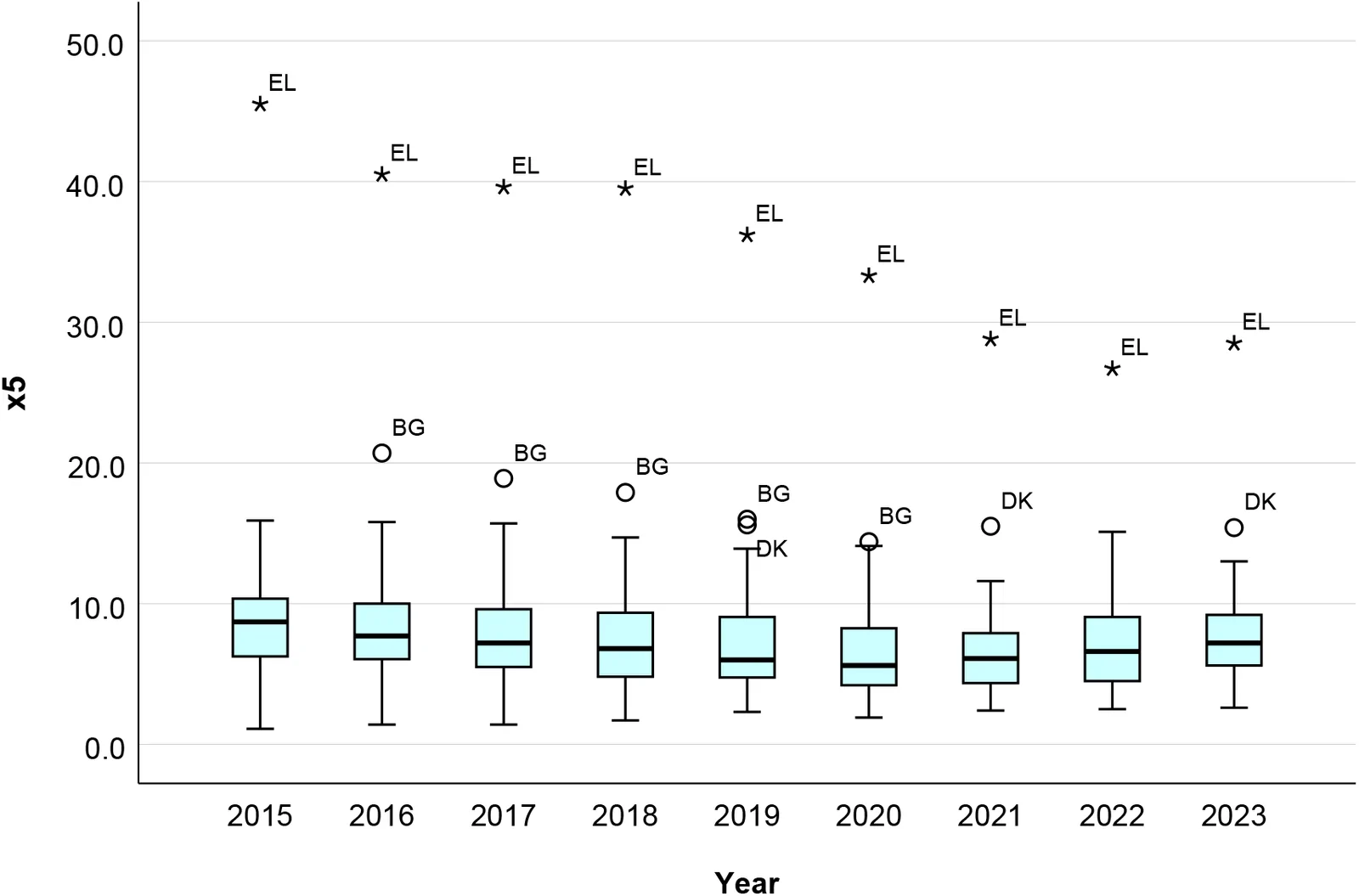
Box plot of housing cost overburden, %. Source: Eurostat, own presentation of *x*_5_.

The severe housing deprivation rate (*x*_6_) was very low in most of the examined countries, the average EU-27 rate was only about 5% in 2013 and it declined to about 4% in 2023. Nonetheless, in some of the countries the rate was higher than 10% and, in some countries, it was even extremely high compared to other countries rates (see Fig 6). The values of indicator *x*_6_ are not available for each year, so the box plots for years 2021 and 2022 are not presented in Fig 6. A significant improvement of the severe housing deprivation rate was reached in Romania where the extremely high rate of 19.8% in 2015 dropped to 9.6% in 2023. A high decline by 10.2 p.p. was achieved also in Hungary, where the rate falls from 15.5% in 2015 to 5.3% in 2023. These changes mark a shift in the desired direction. In 2023 the indicator *x*_6_ was lower than two percent in Malta, Finland, Cyprus, Ireland, the Netherland, Belgium, Denmark and Germany.

**Fig 6:**
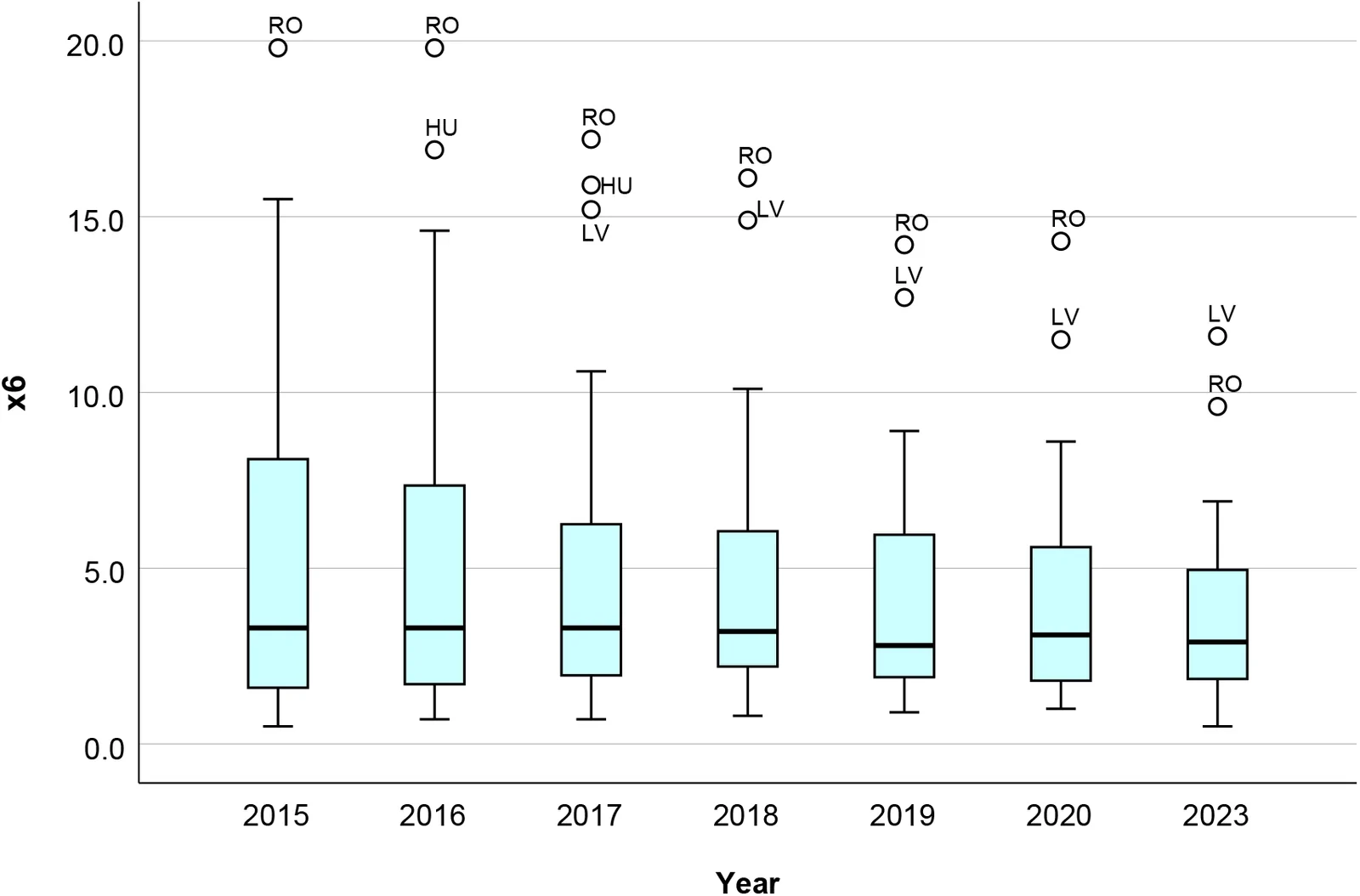
Box plot of severe housing deprivation rate, %. Source: Eurostat, own presentation of *x*_6_.

The aim to analyse the poverty in a broader context meant that not only the indicators from Eurostat to monitor the progress towards the SDG 1 were chosen, but also additional variables were entered into the whole image of the poverty and socio-economic situation in the EU. The indicator that is often used to describe the living standard of the population is GDP per capita in PPS. As it is evident from the box plot presentation in Fig 7, the living standard of the population is increasing except the period of COVID-19 pandemic. The variability of the indicator *x*_7_ was very high especially at the beginning of the examined period. In 2015 the coefficient of variation was as high as 46.6%, the relative variability declined to 38.7% in 2023. Some extreme values of the indicator x_7_ were detected by box plot charts. The extreme values were achieved from 2015 till 2017 in Luxembourg. From 2018 till 2023 two extremely high levels of x_7_ were detected, namely for Ireland and again Luxembourg. The highest relative increase of the indicator *x*_7_ was typical for the Eastern European countries. From 2015 till 2023 the indicator x_7_ increased by 91% in Romania, by 79% in Bulgaria and by 72% in Croatia. These countries achieved the lowest levels of the indicator *x*_7_ in 2015 but the very high increase of the indicator x_7_ denotes a marked increase of the living standard of the population living in the mentioned Eastern European countries.

**Fig 7:**
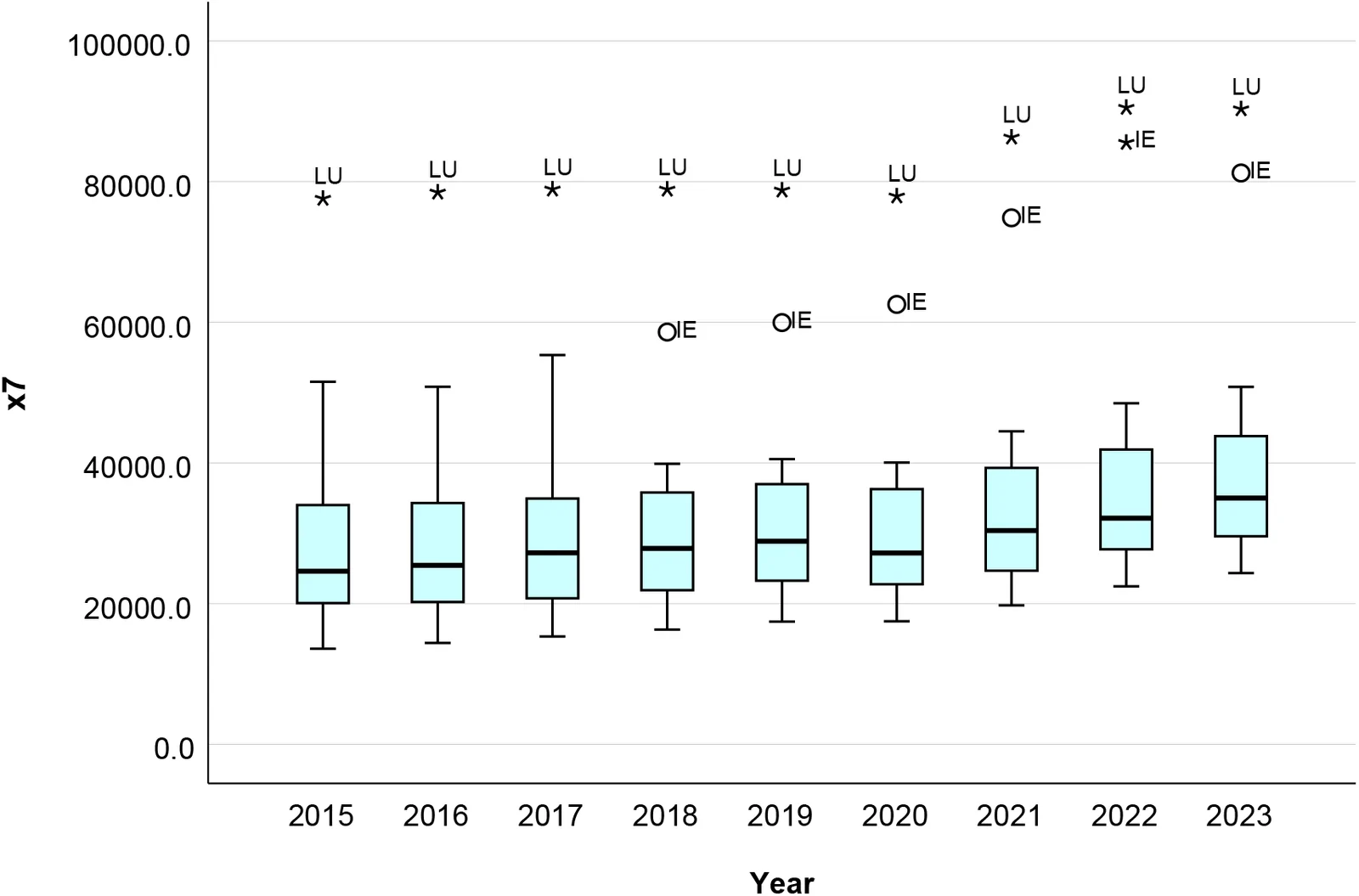
Box plot of GDP per capita, PPS. Source: Eurostat, own presentation of *x*_7_.

Poverty can be overcome through economic growth [13] and therefore also the indicator of real annual GDP growth rate in % (*x*_8_) was included into the overall view of the poverty and socio-economic assessment of countries belonging to the EU. The indicator *x*_8_ remained above zero from 2015 till 2019 (see Fig 8). In 2020 the EU economies were hit by COVID-19 pandemic, and the real GDP shrank in 25 EU countries. The real GDP in 2020 dropped by 10.9% in Spain, by 9.2% in Greece and by 8.9% in Italy. Only in Ireland the indicator *x*_8_ was above zero and as high as 7.2%, in Lithuania the real growth was equal to zero. In 2021 and 2022 a stronger recovery was typical for most of the EU countries. In 2023 the level of x_8_ slowed down, and even the real change was negative in 9 EU countries. An economic downturn can translate later into increased poverty risk.

**Fig 8:**
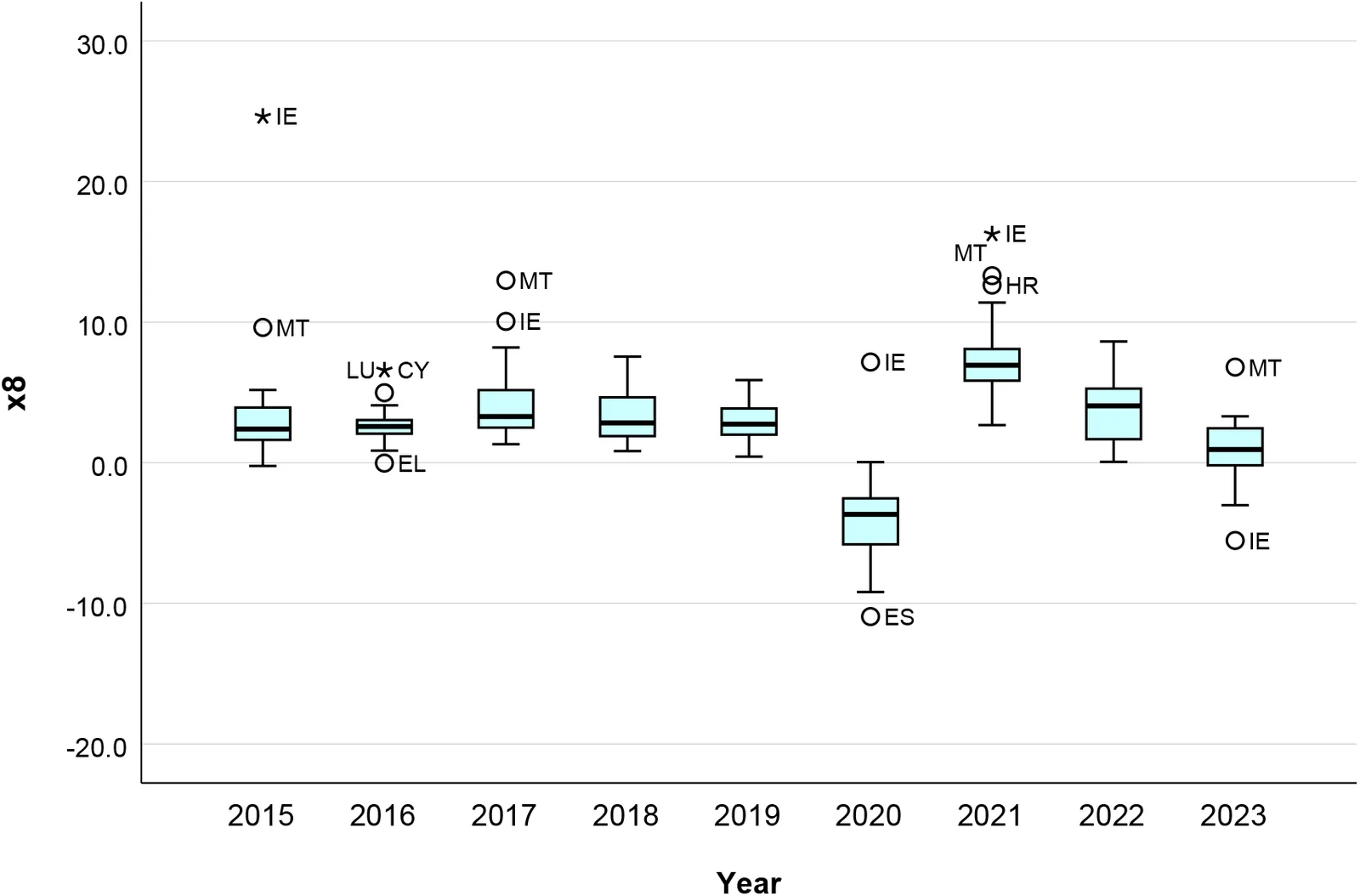
Box plot of real GDP growth rate, %. Source: Eurostat, own presentation of *x*_8_.

The high-speed internet, as a modern technology, is in interest of the EU member states with the ambitious aim to provide a 100-percentage coverage of households with this fixed very high-capacity network by 2030. As the modern technology of high-speed internet has become widespread in recent years, the data for the EU countries are available from 2019 onwards. The variability of the high-speed internet coverage was very high among the EU countries at the beginning of the considered period, and it decreased later slowly (see Fig 9). The importance of the household´s coverage by a high-speed internet was very crucial during and also after the COVID-19 pandemic when a range of activities, including education and the spread of working from home grows. The lack of internet coverage thus hinders progress in studying from home as well as in enabling people to work from home and be sufficiently productive. Eight EU countries had already in 2023, a coverage higher than 90% but, e.g., in Greece it was barely 38.4%. Most of the EU countries will be able to achieve the ambition to increase the coverage by high-speed internet to the expected planned level of 100% but Greece might be an exception.

**Fig 9:**
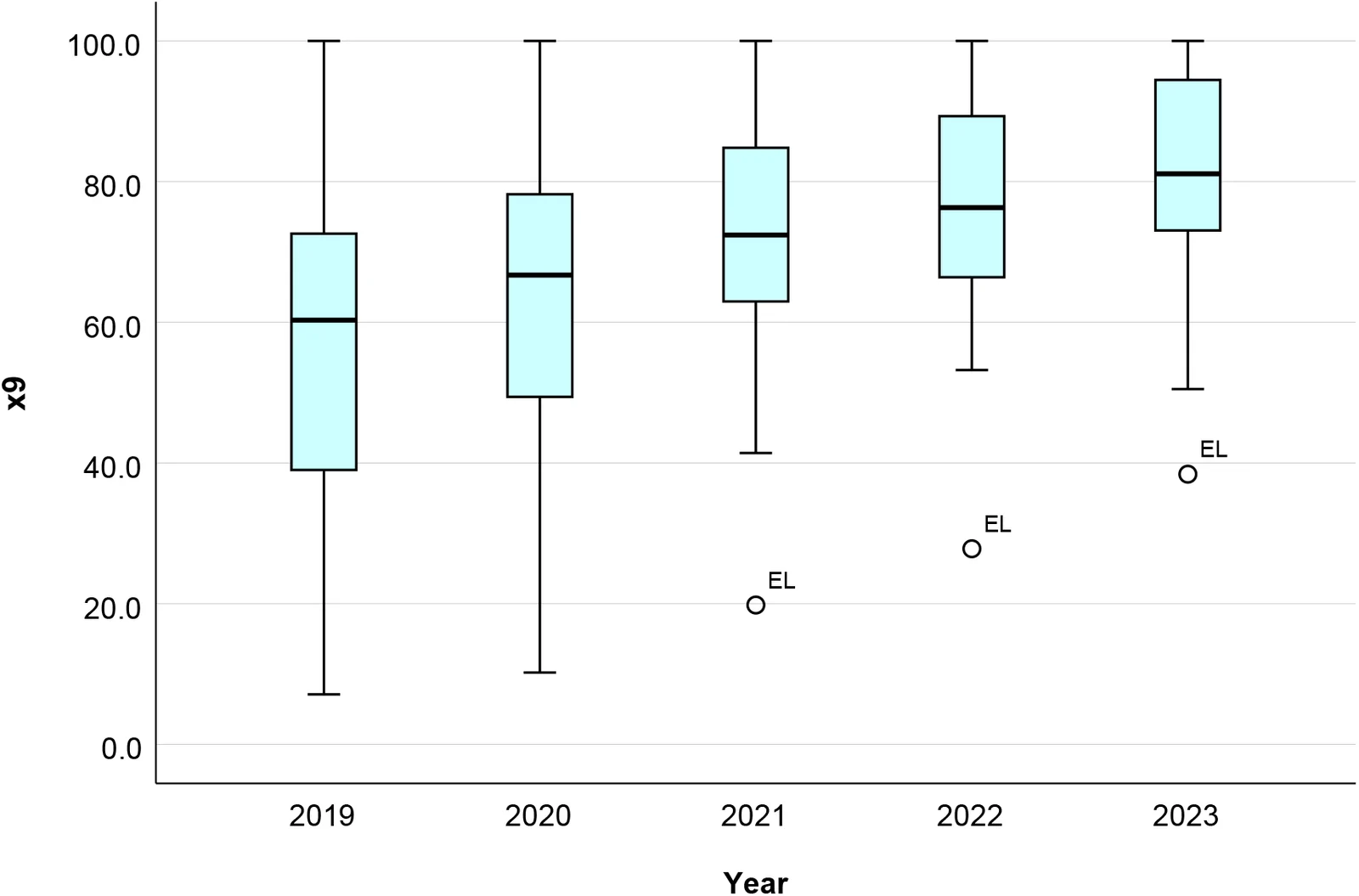
Box plot of high-speed internet coverage, %. Source: Eurostat, own presentation of *x*_9_.

In the study also the share of individuals having at least basic digital skills (*x*_10_) was included as it is expected that only sufficiently educated population in area of information technologies will be properly prepared for the social and economic changes in societies, this knowledge can prevent the population from poverty and secure progress to ensure a competitive edge in the job market. The goal of the EU is to reach a digital decade target by 2030 that is e*x*press by a goal of 80% share of people aged 16–74 that have at least basic digital competences. In 2023 only in the Netherlands and Finland was the threshold of 80% overstepped which is a signal of well-educated and well-prepared working force for the changing world and toward usage of new technologies (see Fig 10). On the other hand, in Romania the share of people with at least basic digital competences in the same year was only 27.7% and in Bulgaria 35.5%. The EU countries should more invest and take more care of lifelong learning of their population in area of digital skills as the digital skills are nowadays, and they will be also in the future very important for keeping the economies growing. The progress in the EU toward this goal is very slow and more economies will have problems to achieve the ambition of the EU to increase the share of *x*_10_ to the expected level of 80%.

**Fig 10:**
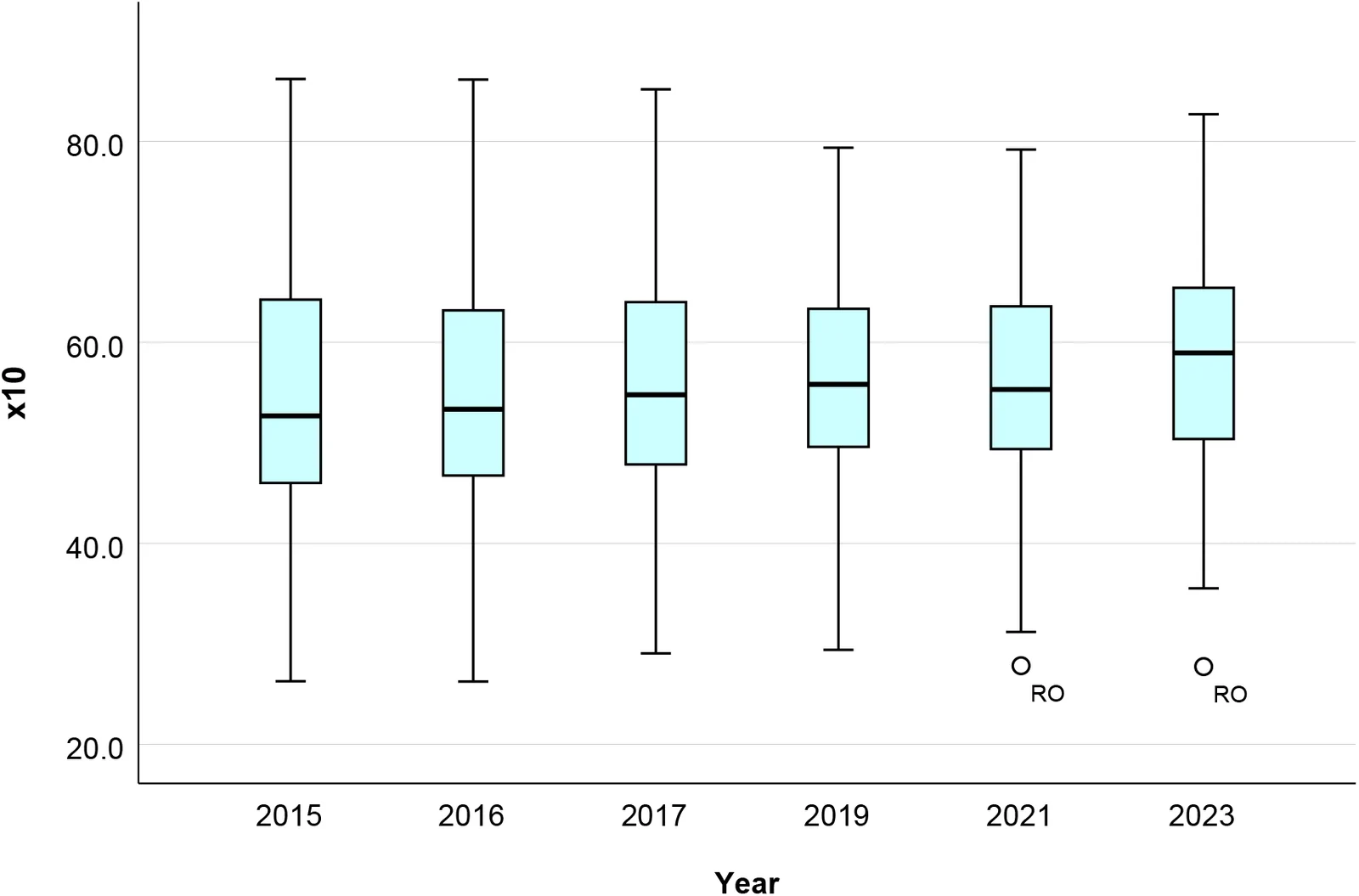
Box plot of the share of individuals having at least basic digital skills, %. Source: Eurostat, own presentation of *x*_10_.

In S3 Table in S1 File, the summary statistics in the three periods can be found.

### 4.1 Corelation analysis and principal components analysis

The input dataset underwent preliminary diagnostic testing before the cluster analysis. The procedures of CA expect uncorrelated dataset and therefore the linear relation between the pairs of variables was checked before clustering. A very strong or strong and statistically significant linear relationship was discovered among more pairs of indicators. These strong correlations were confirmed in all three analysed time spans.

#### 4.1.1 Preliminary diagnostic testing in the first-time span.

In S4 Table the Pearson correlation coefficients of the variables in the first time period are presented (see S1 File). As expected, a strong linear relationship was confirmed between the indicators of poverty. The indicator *x*_1_ was strongly, positively and statistically significantly correlated with the indicators *x*_2_, *x*_4_, *x*_6_. It means that in a country with a high share of persons at risk of monetary poverty also a high percentage of persons severely materially or socially deprived is expected, high rates of the indicator *x*_1_ are expected and also a high rate of severe housing deprivation is a reality and vice versa. On the other hand, the indicator *x*_1_ is negatively, strongly a statistically significantly correlated with the variable *x*_10_ and it means that in countries with high share of persons at risk of monetary poverty a low share of individuals having at least basic skills is supposed to and vice versa. The correlation between the indicators *x*_6_ and *x*_7_ was also negative and strong, in other words in specific countries with a low value of the indicator x_7_, a high level of severe housing deprivation can be presumed. GDP per capita in PPS is strongly, negatively correlated also with the poverty indicator *x*_2_. The share of individuals having at least basic digital skills is negatively and strongly associated with poverty indicators *x*_1_, *x*_2_, *x*_4_, *x*_6_ and positively with the indicator *x*_7_. It can be said that in an EU country with a high share of individuals having at least basic digital skills a lower poverty rate can be foreseen and a higher GDP per capita in PPS was likely to occur. The verification of the strong and statistically significant linear relationship between some pairs of indicators is crucial for the subsequent multivariate analysis. As the CA should be carried out on a dataset where the variables are not correlated before the procedure of cluster analysis, a PCA was done. The main aim of the PCA is to decrease the dataset dimensionality by transforming it into a smaller number of uncorrelated components. Prior the PCA construction the Kaiser´s Measure of Sampling Adequacy (MSA) was examined in each period. For the PCA and for the Kaiser-Meyer-Olkin Test the standardized variables were inputted. The overall MSA (see S5 Table in the Appendix in S1 File) for the 10 input variables reached 0.559 with three indicators having the individual MSA lower than 0.3. These three indicators, namely *x*_3_, *x*_8_ and *x*_9_ were excluded and the Kaiser-Meyer-Olkin Test was repeated with a reduced number of input variables; the overall MSA as the result of the 7 remaining variables increased to 0.691 but the variable *x*_5_ had the individual MSA as low as 0.374 and therefore was also removed from the next step of analysis. The third application of the Kaiser-Meyer-Olkin Test with only 6 input variables brought a satisfactory result as the overall MSA increased to 0.8 and each individual MSA of the six input variables was higher than 0.5 (see S5 Table in S1 File).

Before the clustering procedure of the EU countries, the PCA using six standardised indicators was carried out. The first two principal components accounted for 80.63% of the total variance of 6 input indicators (see S5 Table in S1 File). The standardised variables of indicators *x*_1_, *x*_2_, *x*_4_, and *x*_6_ were strongly and positively associated with the first factor/first principal component, while the indicators *x*_7_ and *x*_10_ were strongly and negatively associated with the same factor. The indicators *x*_4_ and *x*_7_ load highly on factor 2. These two uncorrelated principals were used for the Ward´s hierarchical CA procedure instead of ten original variables.

#### 4.1.2 Preliminary diagnostic testing in the second-time span.

In 2020, a strong and statistically significant linear relation was observed between certain indicators (see S6 Table in S1 File) and therefore the PCA was performed again. In the first step the Kaiser-Meyer-Olkin Test of 10 standardized variables in the second-time span was performed. Similarly to the first-time span, in 2020, three indicators also had the individual MSA (see S7 Table in S1 File) at a low level, which resulted in the exclusion of these variables *x*_3_, *x*_8_ and *x*_9_ from the next stage of analysis. The Kaiser-Meyer-Olkin Test was repeated with seven variables left; the overall MSA increased from 0.601 to 0.724 but still one variable had the individual MSA as low as 0.38. The exclusion of the indicator *x*_5_ resulted in an increase of the overall MSA to 0.791 and each of the individual MSA was higher than 0.5. In the next stage of the analysis only six standardised variables were used for the PCA. In this solo year 2020 first two extracted components explained 79.72% of the total variance (see S7 Table in S1 File) and these two principals were used for clustering. Very similarly to the first-time span also in the second period the first principal component explained a large portion of the variance in indicators *x*_1_, *x*_2_, *x*_4_, *x*_6_, *x*_7_, *x*_10_. The second factor was strongly and positively associated with the indicators *x*_4_ and *x*_7_. The cluster analysis was performed using the two uncorrelated principal components instead of ten original input variables.

#### 4.1.3 Preliminary diagnostic testing in the third-time span.

Similar to the correlation analysis in the first and second periods, also in the third-time span, the correlation among some pairs of indicators was strong and statistically significant (see S8 Table in S1 File). The Pearson correlation coefficient between the variables *x*_1_ and *x*_4_ was as high as 0.75, so in a country with a high percentage of persons at risk of monetary poverty after social transfers a high share of persons who are employed and have an equivalised disposable income below the risk-of-poverty threshold can be expected and vice versa. On the other hand, the correlation between the variables *x*_6_ and *x*_10_ was, for example, high and negative (*r*_*xy*_ = −0.68) and it can be explained that in the countries with a high share of people aged 16–74 who have at least basic digital skills a lower severe housing deprivation rate is expected. Due to the fact that some pairs of indicators are strongly or moderately correlated, the PCA was implemented before the multivariate CA. According to the results of the Kaiser-Meyer-Olkin Test of 10 standardized variables in the third-time span the overall MSA reached 0.599 but the individual MSA was lower than 0.4 for four variables (see S9 Table in S1 File). After the elimination of the four indicators with the lowest individual MSA, the Kaiser-Meyer-Olkin Test was repeated. The overall MSA increased to 0.761 and each of the six remaining indicators had an individual MSA higher than 0.5. The principal component analysis was accomplished using only the six standardised variables like in the first- and second-time spans. Based on the PCA results, presented in S9 Table (S1 File), the first two most important principal components that accounted for 76.81% of the total variance of six input variables were chosen and used for clustering. The standardised variables of indicators *x*_1_, *x*_2_, *x*_4_, and *x*_6_ were strongly and positively associated with the first principal component, while the indicators *x*_7_ and *x*_10_ were strongly and negatively associated with it.

### 4.2 Cluster analysis

The cluster analysis in each of the three time periods was performed using the most important principal components as the result of the analysis in subchapter 4.1.

#### 4.2.1 Cluster analysis – first-time span: years 2015–2019.

The dendrogram of the clustering in the first-time span is presented in the S1 Fig in S1 File. The aim was to split the EU countries into at least three and at most ten clusters so that the created clusters allow to make a deeper analysis of the EU countries classified into a specific cluster. It was expected that, from the perspective of the indicators used for clustering, the resulting clusters would exhibit a weak to moderate cluster structure and might overlap to some extent, while still supporting a meaningful clustering decision. For a correct decision on the number of clusters not only the Silhouette statistics but also a visual inspection of the dendrogram was used. The highest Silhouette in the first-time span was 0.39 dividing the EU countries into ten clusters with two solo clusters, four clusters with only two countries and other clusters with three, four or six countries. This solution wat not considered as the best one since one individual Silhouette for a concrete cluster was negative. The EU countries were after the inspection of the Silhouette divided into six clusters, the Silhouette for this solution was 0.34 (see S10 Table in S1 File) and it indicates a weaker to moderate cluster structure with still a useful and meaningful decision. More detailed description of the clusters was done using the cluster centroids presented in Table 1. The cluster centroids were defined for the standardized indicators as well as for the original input variables; the cluster centroids of the standardized and original indicators are presented for a better understanding of the values of analysed variables.

**Table 1:** Cluster centroids of the EU countries (first-time span).

Clusters of EU Countries	Cluster 1	Cluster 2	Cluster 3	Cluster 4	Cluster 5	Cluster 6	Total_EU
**No. of Countries**	**8**	**5**	**6**	**6**	**1**	**1**	**27**
**Zx1_2015–2019**	−0.821	0.510	−0.587	0.961	−0.118	1.899	0.00
**Zx2_2015–2019**	−0.426	0.750	−0.612	0.191	−0.843	3.025	0.00
**Zx4_2015–2019**	−0.840	0.110	−0.459	0.857	0.975	2.804	0.00
**Zx6_2015–2019**	−0.594	1.282	−0.621	−0.012	−0.657	2.789	0.00
**Zx7_2015–2019**	−0.107	−0.831	0.792	−0.433	3.734	−0.873	0.00
**Zx10_2015–2019**	0.126	−0.869	0.891	−0.308	1.889	−2.047	0.00
**x1_2015–2019**	13.6	18.8	14.5	20.6	16.3	24.3	16.8
**x2_2015–2019**	5.0	13.9	3.6	9.7	1.9	31.0	8.2
**x4_2015–2019**	5.6	8.6	6.8	11.0	11.4	17.2	8.3
**x6_2015–2019**	2.3	10.7	2.2	4.9	2.1	17.4	5.0
**x7_2015–2019**	28478.4	19052.2	40197.0	24237.8	78517.1	18510.2	29878.7
**x10_2015–2019**	57.2	44.1	67.3	51.5	80.5	28.5	55.6

Source: Eurostat, own calculations

*Cluster 1 (Belgium, Czechia, France, Cyprus, Malta, Slovenia, Slovakia, Finland).* Mostly “newer” EU member states together with Belgium, France and Finland joined together into the cluster 1. These countries had achieved a minimal value of shares of persons at risk of monetary poverty and of the share of persons who are employed and have an equivalised disposable income below the risk-of-poverty threshold (thus the in-work at risk-of-poverty rate). They performed very good also in terms of low levels of severe housing deprivation rate, severe material and social deprivation rate, their GDP per capita in PPS was very similar to the EU-27 average and the share of people aged 16–74 who have at least basic digital skills was close to the EU average as well. These countries achieved strong performance on the poverty indicators while attaining average levels on the living standards and digital skills indicators.

*Cluster 2 (Bulgaria, Croatia, Latvia, Hungary, Poland).* The cluster with only “newer” member states had the second lowest GDP per capita in PPS. Their position among the clusters was the second worst regarding to the share of people who have at least basic digital skills, severe housing deprivation rate, severe material and social deprivation rate. These countries can be rated negatively in the context of lower living standard with combination of high rates of the poverty indicators.

*Cluster 3 (Denmark, Germany, Ireland, the Netherlands, Austria, Sweden).* The well developed “older” EU nations grouped within the cluster 3. The countries had the second highest value of GDP per capita in PPS and the second highest share of people who have at least basic digital skills. They performed very well in achieving the second-best positions in poverty indicators (share of persons at risk of monetary poverty, severe material and social deprivation rates, in-work at risk-of poverty rate and severe housing deprivation rate). The cluster 3 can be rated as the best cluster with a combination of a very high living standard, very low levels of the poverty indicators and high percentage of population that have at least basic digital skills.

*Cluster 4 (Estonia, Greece, Spain, Italy, Lithuania, Portugal).* The PIGS countries (Portugal, Italy, Greece, Spain) and two Baltic countries joined together into the cluster 4. The second worst position reached these countries in share of persons at risk of monetary poverty and very high was also the share of persons who are employed and have an equivalised disposable income below the risk-of-poverty threshold. The severe housing deprivation rate in cluster 4 was nearly at the EU-27 average. The GDP per capita in PPS and the share of people who have at least basic digital skills are in the middle of the clusters, so the biggest problem of the six countries in cluster 4 are the high levels of the poverty indicators.

*Cluster 5 (Luxembourg).* Luxembourg did not cluster with any other EU country and therefore formed a separate cluster. According to the cluster centroids (see Table 1), Luxembourg achieved the highest GDP per capita in PPS and the highest share of people who have at least basic digital skills. The country also performed best in terms of severe material and social deprivation and severe housing deprivation. However, the country did not perform well with respect to the in-work at risk-of-poverty rate, which was the second highest among the analysed countries, while the share of persons at risk of monetary poverty was also relatively high. Thus, although the country performed very well in terms of living standards and digital skills of the population, the levels of the poverty indicators revealed a mixed picture, suggesting that high economic performance does not necessarily translate into favourable outcomes across all dimensions of poverty.

*Cluster 6 (Romania).* The second solo cluster was created by Romania. This cluster was the worst one compered to others. Each indicator of Romania achieved the poorest results in comparison with other clusters or countries. Very high were the poverty variables indicating huge destitution problems of the population and on the other hand very law was the GDP per capita in PPS and very low was the share of people who have at least basic digital skills. The combination of the very negative results in each indicator resulted in the Romania´s separation from other EU countries.

Overall, the results for the first period showed that in some clusters only “older” more developed EU countries were joined together (cluster 3, cluster 5), whereas others are created by only “newer” and economically less developed Member States (cluster 2, cluster 6). Clusters 1 and 4 included a mixture of older and newer Member States indicating similarities in their poverty indicators, socio-economic indicators, digital development variable. The diagram of the classification of countries into individual clusters is illustrated in S2 Fig in S1 File.

#### 4.2.2 Cluster analysis – second-time span: year 2020.

In the second time span the EU countries were divided into five clusters using the Silhouette statistics and inspection of the dendrogram (see S11 Table in S1 File and S3 Fig in S1 File). As in the first time span also in the second period the Silhouette was not higher than 0.5, it achieved a value of 0.33 that still indicate a useful and meaningful decision with a possibility of overlapping clusters and a weaker to moderate cluster structure of the EU countries. The cluster centroids of the EU countries in the second period can be found in Table 2.

**Table 2:** Cluster centroids of the EU countries (second-time span).

Clusters of EU Countries	Cluster 1	Cluster 2	Cluster 3	Cluster 4	Cluster 5	Total_EU
**No. of Countries**	**9**	**2**	**6**	**9**	**1**	**27**
**Zx1_2020**	−0.772	1.895	−0.513	0.659	0.306	0.00
**Zx2_2020**	−0.291	2.953	−0.468	0.035	−0.801	0.00
**Zx4_2020**	−0.870	1.593	−0.439	0.645	1.467	0.00
**Zx6_2020**	−0.265	2.204	−0.653	0.297	−0.780	0.00
**Zx7_2020**	−0.270	−0.880	0.767	−0.438	3.533	0.00
**Zx10_2021**	0.146	−2.213	0.984	−0.379	0.619	0.00
**x1_2020**	13.2	23.6	14.2	18.8	17.4	16.2
**x2_2020**	4.7	23.7	3.7	6.6	1.7	6.4
**x4_2020**	5.4	12.3	6.6	9.6	11.9	7.8
**x6_2020**	3.3	11.5	2.0	5.2	1.6	4.2
**x7_2020**	27756.6	19708.7	41436.4	25535.6	77913.1	31317.7
**x10_2021**	58.1	29.5	68.2	51.7	63.8	56.3

Source: Eurostat, own calculations

*Cluster 1 (Belgium, Czechia, France, Croatian, Cyprus, Hungary, Slovenia, Slovakia, Finland).* In cluster 1 altogether nine countries linked together. This larger cluster consists of three older and six newer EU member states. Best results achieved these countries in two indicators, namely the percentage of persons at risk of monetary poverty and the in-work at risk-of-poverty rate were minimal in the cluster 1. Good results were reached in other poverty indicators too. Slightly above the EU average resulted the share of people who have at least basic digital skills and the GDP per capita in PPS was higher than in clusters 2 and 4. The cluster 1 must be positively rated especially due its low poverty rates while other analysed variables reached satisfactory levels.

*Cluster 2 (Bulgaria, Romania).* In the second period Romania joined together with Bulgaria. These two economically less developed countries formed the worst cluster as each of the analyzed indicators reached in this cluster their worst levels. The populations of Bulgaria and Romania were the poorest compared with those of other EU countries, as the poverty indicators for cluster 2 reached the highest values and the GDP per capita in PPS was the lowest, as was the share of people with at least basic digital skills. The outcome of the selected variables in cluster 2 must be rated very negatively.

*Cluster 3 (Denmark, Ireland, Malta, the Netherlands, Austria, Sweden).* From the six countries that formed the cluster 3 only Malta belong to the “newer” member states of the EU. These countries had the second highest value of GDP per capita in PPS and the highest share of people who have at least basic digital skills. They performed well in achieving very low values of the poverty variables that indicate a very good conditions for a high quality of life. This cluster can be rated as the best one compared to other clusters. In comparison with cluster 5, very positively must be rated the low in-work at risk-of-poverty rate that achieved in cluster 3 only 6.6% while in cluster 5 it was as high as 11.9%. Especially the combination of a very high living standard, very low levels of the poverty indicators and high percentage of population that have at least basic digital skills shifts this cluster to the best one.

*Cluster 4 (Germany, Estonia, Greece, Spain, Italy, Latvia, Lithuania, Poland, Portugal).* As in cluster 1 also in cluster 4 nine countries joined together. These countries represent a mix of older and newer member states. Countries in cluster 4 had the second highest levels of share of persons at risk of monetary poverty and severe material and social deprivation rate. The share of people who have at least basic digital skills and the GDP per capita in PPS were below EU averages. The countries in cluster 4 did not perform very well regarding to the analysis variables.

*Cluster 5 (Luxembourg).* The only solo cluster was built by Luxembourg. The population in this country had the minimal severe material and social deprivation rate and minimal severe housing deprivation rate in combination with the highest GDP per capita in PPS. Very positive was also the second-best share of people who have at least basic digital skills however the share declined compared to the first-time span. Negatively must be rated the very high share of persons who are employed and have an equivalised disposable income below the risk-of-poverty threshold which was the second worst. High was also the percentage of persons at risk of monetary poverty after social transfers. Luxembourg reached the best results of 3 indicators and very bad results of 2 indicators that was an unusual combination; this outcome is like the result from the first analysed period.

The results of the cluster analysis for the second period, which was affected by the COVID-19 pandemic, discovered a greater mixture of older and newer EU countries within the same clusters (see S4 Fig in S1 File). Only cluster 2 was composed by two less developed newer EU member states (Bulgaria, Romania), while a single-country cluster 5 was represented only by Luxembourg. This analysis outcome in the second-time span revealed mixing the “older” and “newer” EU member states into common clusters which means that they have similarities with respect to the selected indicators.

#### 4.2.3 Cluster analysis – third-time span: years 2021–2023.

The Silhouette statistics as well as the dendrogram (see S5 Fig in S1 File) of the clustering in the third-time span can be found in the Appendix. The EU countries were after the inspection of the Silhouette divided into seven clusters, the Silhouette for this solution reached 0.40 (see S12 Table in S1 File) and it indicate a weaker to moderate cluster structure with still a useful and meaningful decision. The split of the EU countries into seven clusters resulted in creation of two solo clusters, one cluster with two objects and four clusters with four or more object. The more details description of the clusters using the cluster centroids (see Table 3) is presented below.

**Table 3:** Cluster centroids of the EU countries (third-time span).

Clusters of EU Countries	Cluster 1	Cluster 2	Cluster 3	Cluster 4	Cluster 5	Cluster 6	Cluster 7	Total_EU
**No. of Countries**	**8**	**4**	**2**	**6**	**5**	**1**	**1**	**27**
**Zx1_2021–2023**	−0.573	1.345	−1.475	−0.397	1.000	−0.940	0.476	0.00
**Zx2_2021–2023**	−0.210	1.852	−0.775	−0.476	−0.094	−0.120	−0.729	0.00
**Zx4_2021–2023**	−0.439	1.113	−1.642	−0.393	0.750	−0.943	1.894	0.00
**Zx6_2023**	−0.131	1.789	−0.761	−0.666	0.194	−0.951	−0.609	0.00
**Zx7_2021–2023**	−0.378	−0.818	−0.121	0.398	−0.398	2.796	3.344	0.00
**Zx10_2023**	−0.319	−1.373	1.417	0.697	−0.074	1.208	0.199	0.00
**x1_2021–2023**	14.1	21.3	10.7	14.8	20.0	12.7	18.1	16.3
**x2_2021–2023**	4.9	15.3	2.1	3.6	5.5	5.4	2.3	6.0
**x4_2021–2023**	6.7	11.4	3.0	6.8	10.3	5.1	13.7	8.0
**x6_2023**	3.5	8.5	1.8	2.1	4.3	1.3	2.2	3.8
**x7_2021–2023**	31311.1	24501.0	35305.1	43345.9	31013.6	80530.9	89036.2	37178.3
**x10_2023**	53.6	40.2	75.6	66.4	56.7	72.9	60.1	57.6

Source: Eurostat, own calculations

*Cluster 1 (Belgium, France, Croatia, Cyprus, Hungary, Poland, Slovenia, Slovakia).* The largest cluster in the third period was created by eight countries from which most belong to the “newer” EU members. The share of people who have at least basic digital skills was the second lowest among clusters which in time of the growing need to master computer skills is not a good performance. These countries need to invest more time, money and effort for education to increase the digital skills of their population. The levels of the poverty indicators were better compared with clusters 2 and 5 and in case of the indicators in-work at risk-of-poverty rate and share of persons at risk of monetary poverty they performed better also compared with the cluster 7. The countries in cluster 1 can be rated as average regarding to some of the analysed indicators.

*Cluster 2 (Bulgaria, Greece, Latvia, Romania).* Bulgaria and Romania were together in a common cluster also in the second-time span and now, in the third-time span, they were linked together with Latvia and Greece. For the countries in this cluster was typical a low GDP per capita in PPS with a combination of the highest share of persons at risk of monetary poverty, highest severe material and social deprivation rate, highest severe housing deprivation rate and the second highest in-work at risk-of-poverty rate. The share of people who have at least basic digital skills was as low as 40.2%. Similarly to the cluster 1 these countries also need invest more time, effort and support for the education of population to increase its digital skills. Cluster 2 was rated as the worst one among all cluster in the third-time span.

*Cluster 3 (Czechia, Finland).* Only two countries linked together into the cluster 3. These countries achieved very good results regarding to the poverty indicators. Lowest were the share of persons at risk of monetary poverty, severe material and social deprivation rate, in-work at risk-of-poverty rate and second lowest was the severe housing deprivation rate. Their population reached the heist share of people aged 16–74 who have at least basic digital skills. All these very good results of the mentioned indicators with a GDP per capita in PPS near to the EU average shift the cluster 3 to the best one.

*Cluster 4 (Denmark, Germany, Malta, the Netherlands, Austria, Sweden).* The composition of countries in cluster 4 in the third-time span and cluster 3 in the second-time span are very similar. These countries achieved a high value of GDP per capita in PPS especially if we do not consider the extremely high GDP per capita values of Ireland and Luxembourg in two solo clusters. These countries performed well also in terms of poverty indicators which were in the middle among the clusters. The share of people who have at least basic digital skills was lower than in cluster 3 and 6 but higher compared to clusters 1, 2, 5 and 7. Results of the analysed indicators in the cluster 4 can be rated positively.

*Cluster 5 (Estonia, Spain, Italy, Lithuania, Portugal).* Countries in the cluster 5 had the second lowest GDP per capita in PPS, the second highest share of persons at risk of monetary poverty, severe material and social deprivation rate and the in-work at risk-of-poverty rate. The combination of the low GDP per capita in PPS with the high levels of poverty indicators resulted in a negative evaluation of the countries in cluster 5.

*Cluster 6 (Ireland).* Ireland like Luxembourg appeared separately in a solo cluster. Ireland had the lowest sever housing deprivation rate, the second lowest share of persons who are employed and have an equivalised disposable income below the risk-of-poverty threshold, the second lowest was also the share of persons at risk of monetary poverty after social transfers. The second highest was its GDP per capita in PPS and the share of people who have at least basic digital skills. Overall, this cluster can be rates as the best one especially due to a high level of GDP per capita with a combination with a very low levels of the poverty indicators and a high share of population who have at least basic digital skills.

*Cluster 7 (Luxembourg).* Luxembourg as in the first- and the second-time spans again did not join with any other EU country. Cluster 7 achieved the highest GDP per capita in PPS that indicate a high living standard, but this extremely high GDP per capita was combined with the highest in-work at risk-of-poverty rate. High was also the share of persons at risk of monetary poverty after social transfers. In comparison with the solo cluster 6 especially some of the poverty indicators indicate a worse situation regarding the poverty reduction.

In the third time span, the worst-rated cluster, comprising Bulgaria, Greece, Latvia and Romania, achieved poor results for all indicators used for clustering. Two solo clusters were not joined together especially due to the different situation in both countries regarding to a different level of their poverty indicators and a high disparity of their share of people who have at least basic digital skills. The best-rated clusters were the single-country cluster 6 (Ireland) and the cluster 3 (Czechia, Finland). S6 Fig in S1 File shows the diagram of the classification of countries into the individual clusters.

## 5 Discussion and conclusion

The study presents outcomes on the alleviation of poverty, consistent with the results of research on the relation between the SDGs and digital transformation [61]. Concurrently it confirms the existence of persistent regional disparities across EU Member States. It also points to structural weaknesses as well as forms of resilience within individual EU countries. The recommendations can be framed on two levels. First, cross-cutting measures applicable to all member states, namely adequate minimum-income schemes [62] and child-benefit coverage, housing affordability and energy-efficiency upgrades, measures to improve job quality; and investment in digital access and skills, in line mostly with the 2030 Agenda [63] and the European Pillar of Social Rights Action Plan [64]. Funding and implementation can be further supported, where applicable, through the Recovery and Resilience Facility, the Social Climate Fund and the Digital Decade Policy programme 2030 [65], and the European Social Fund Plus (ESF+) [66]. Second, targeted interventions should be tailored to the specific socio-economic profiles of individual clusters.

Since the third period captures the most recent socio-economic situation and produces the highest degree of cluster differentiation, the following discussion is primarily based on the cluster structure identified in this period. The results from the first and second periods are subsequently used to assess the stability of country positions and the dynamics of cluster transitions over time.

Bulgaria, Romania, Greece, and Latvia form the most vulnerable cluster in the final period, reflecting persistent multidimensional deprivation and structural challenges. As reported by Sedefoğlu and Dudek [18], Bulgaria and Romania were among the lowest-ranked cases in Europe over 2015–2022. Likewise, positional TOPSIS results kept Bulgaria and Romania in the persistent severe poverty bracket in 2018 [41], while Greece and Latvia also exhibited elevated poverty levels, although with different levels of intensity. In the case of Greece and Latvia, the final-period cluster membership indicates increasing similarities in poverty, housing and digital indicators, suggesting a convergence towards a common vulnerability profile. Although Greece recorded the highest economic growth among analysed EU countries in the last period, this growth is mainly the output of a previous sharp decline. The government has announced further tax relief and pension increases, as well as broader tax breaks and family-oriented measures [67,68]. All these countries are experiencing high economic growth and gradual improvement in the area of housing deprivation; however, they still lag significantly behind the rest of Europe, mainly in the area of digital competences. This points to the necessity of sustained and targeted investment in infrastructure, public service, residential policy, and internet accessibility in order to reduce the deep disparities with other Member States. Bridging this gap requires institutional alignment, advocacy coalitions, and budgetary anchoring across European semester cycles. Persistent pockets of deprivation and ongoing cost-of-living pressures in these countries leave them highly exposed to external shocks.

Belgium, France, Croatia, Cyprus, Hungary, Poland, Slovenia and Slovakia form a relatively stable and balanced socio-economic cluster in the final period. Despite differences in their levels of economic development, these countries exhibit similar multidimensional poverty profiles, suggesting that economic performance alone does not fully explain social vulnerability within the EU [69]. Their progress is visible, while relatively favourable values of poverty, material deprivation and housing indicators contribute to their cluster membership. Cyprus and Croatia achieved substantial GDP growth between 2021 and 2023 thanks to the recovery of tourism after the restrictions during the Covid-19 pandemic. Complementary evidence from a separate EU-wide analysis of material and social deprivation points to an improvement in Cyprus and, at variance with our results, in Slovenia [18]. The convergence of these countries into a common cluster highlights the importance of considering social, housing and digital dimensions simultaneously, rather than relying solely on economic indicators such as GDP per capita.

A particular interesting result emerged in the final period, when the Czech Republic and Finland formed a separate cluster characterised by very favourable social and digital indicators. The cluster is distinguished by low levels of poverty, material deprivation, in-work poverty and housing deprivation, together with high levels of digital skills. This finding suggests that multidimensional poverty profiles are influenced not only by economic performance, but also by social resilience and digital preparedness.

A set of Western and Northern European countries (Denmark, Germany, the Netherlands, Austria and Sweden), together with Malta, shows relative stability and low levels of multidimensional poverty, which corresponds with similar studies [61,70], according to which the most developed economies are leading the way in achieving the SDGs. These countries are characterised by high GDP per capita, favourable digital indicators and relatively low levels of poverty and deprivation, confirming their strong socio-economic resilience.

Ireland formed a separate cluster in the final period, reflecting its specific socio-economic profile. Very high GDP per capita and favourable digital indicators distinguish the country from the remaining Member States, indicating that its development trajectory differs from both the economically advanced Western countries and the socially resilient Czech-Finnish cluster.

Particular attention should be paid to Luxembourg, where an economic paradox emerges. Although the country is rated very favourably overall (mainly due to its high GDP/capita in PPS), selected indicators show results that signal internal imbalances, e.g., *x*_1_ increased between 2015 and 2023. In Luxembourg, housing costs are among the highest in the EU (about 86% above the EU average in 2023), and housing prices are still rising [71], which indicates that high economic performance does not necessarily translate into good social outcomes in all dimensions. Policy efforts should therefore prioritise housing affordability and cost containment.

Spain, Italy, Portugal, Lithuania, and Estonia consistently belonged to the same cluster through all analysed periods, indicating a stable multidimensional poverty profile. Although these countries do not belong to the most vulnerable group, they exhibit higher levels of poverty risk, in-work poverty and housing deprivation than the more resilient clusters. Their economic performance and digital indicators remain at intermediate levels, placing them between the most vulnerable and the most prosperous group of countries. The persistent character of this cluster suggests the existence of the long-term structural challenges related to poverty, housing conditions and labour market outcomes. Also, the inclusion of both Southern European and Baltic countries indicates that multidimensional poverty profiles cannot be explained solely by geographical location or economic development. Instead, similar combinations of social, housing and digital characteristics appear to shape the observed clustering patterns.

Considering time trends, the comparison of the three analysed periods indicates that while several countries changed their cluster membership, a number of stable multidimensional profiles persisted throughout the analysed period. Belgium, France, Cyprus, Slovenia and Slovakia exhibited a particularly high degree of stability, remaining in the same cluster throughout all analysed periods. Similarly, Denmark, the Netherlands, Austria and Sweden formed a stable group of countries characterised by favourable economic and digital conditions. Luxembourg maintained a highly specific position and repeatedly formed a separate cluster, reflecting its unique combination of exceptionally high economic performance and distinctive social characteristics.

On the other hand, several countries experienced more pronounced changes in cluster membership. Romania moved from an isolated position in the first period towards a common vulnerability cluster with Bulgaria and, in the final period, also with Greece and Latvia, suggesting increasing similarities in multidimensional deprivation patterns. Greece gradually shifted from a broader Southern European cluster towards a more vulnerable group of countries. Conversely, Hungary and Poland converged towards a balanced socio-economic cluster together with Belgium, France, Slovakia and Slovenia. The final period also revealed two distinctive cases – a separate Czech-Finnish cluster characterised by very favourable social and digital indicators, and an individual cluster of Ireland reflecting its specific economic and digital profile. Overall, the findings indicate that multidimensional poverty profiles evolve over time and cannot be explained solely by geographical location or the level of economic development.

The identified country clusters also delineate distinct operating conditions for enterprises. In clusters with high levels of poverty, constrained household budgets, excessive housing cost burden and lower digital readiness translate for a corporate sector into more fragile and less predictable local demand, greater customer price sensitivity and a higher risk of payment difficulties and bad debts. At the same time, labour markets in these contexts are often characterised by lower skill levels and greater employment instability, which is reflected in more demanding recruitment processes, higher requirements for internal training, higher staff turnover and volatile productivity. By contrast, clusters with lower multidimensional poverty and stronger digital infrastructure provide more favourable conditions for productivity-enhancing investment and the deployment of green and digital technologies. Understanding these territorial patterns is therefore relevant not only for the design of social policies, but also for corporate strategies in areas such as market segmentation, decisions on where enterprises operate, human resource management and ESG-oriented business models.

From a methodological point of view, correlations between several poverty indicators have been demonstrated. It is necessary to highlight the indicator: persons at risk of monetary poverty after social transfer (*x*_1_) which correlates positively with the indicators severe material and social deprivation rate (*x*_2_), in work at-risk-of-poverty rate (*x*_4_), and severe housing deprivation rate (*x*_6_). A negative correlation was confirmed between the indicator share of individuals having at least basic digital competences (*x*_10_) and several poverty indicators: *x*_1_, *x*_2_, *x*_4_, and *x*_6_. The confirmation of the key role of digital technologies in alleviating poverty and reducing inequalities is also consistent with results of other research [61]. Our findings also confirm the need to monitor hidden indicators of poverty related to housing. However, not all selected indicators contributed equally to the common multidimensional poverty structure. The exclusion of persons living in households with very low work intensity (*x*_3_), the housing cost overburden rate (*x*_5_), annual real GDP growth rate (*x*_8_), and high-speed internet coverage (*x*_9_) during the PCA-based variable selection indicates that these variables did not share sufficient common variance with the remaining indicators. The exclusion of the indicator representing persons living in households with very low work intensity suggests that household work intensity reflects a distinct aspect of socio-economic vulnerability that is not fully captured by the remaining poverty indicators. The exclusion of *x*_5_ indicates that housing affordability represents a specific dimension of vulnerability that cannot be explained solely by income poverty or material deprivation and may therefore require dedicated policy measures. The exclusion of *x*_8_ from the common factor structure suggests that short-term macroeconomic fluctuations are not closely aligned with the common multidimensional poverty structure across EU Member States. Finally, the exclusion of *x*_9_ indicates that digital infrastructure coverage, unlike digital skills (*x*_10_), does not sufficiently differentiate multidimensional poverty patterns, which may suggest that broadband coverage is less informative for differentiating multidimensional poverty patterns than digital skills.

Of particular interest were the results related to the integration of GDP per capita in PPS – in some instances, inverse correlations with selected indicators were found, but positive correlations with the digital skills indicator were confirmed in all periods. This indicator thus provided additional value in interpreting the results.

Limitations of this study include the time horizon (2015–2023), so the findings may not fully capture earlier structural trends or post-2023 developments. The results may also be influenced by other factors, such as the type and composition of households [10], which were not fully observed in this paper. Moreover, future studies could extend the scope of the research by examining poverty in relation to qualification level, health status of the population [13], and in a broader context that includes energy poverty. Recent evidence confirms that energy poverty is closely associated with income inequality and long-term unemployment, suggesting that economic growth alone is insufficient to mitigate it [38]. In addition, studies applying multidimensional and factor-analytic approaches have demonstrated significant disparities in both the extent and intensity of energy poverty across European countries, with Greece, Lithuania, and Bulgaria being the most affected, whereas Ireland shows relatively higher targeting efficiency [72].

Further research could build on these findings by exploring how socio-economic factors, regional disparities and household characteristics interact to shape vulnerability and access to essential resources within the EU context. In line with prior work [73], a human-rights framing has symbolic and mobilising force, yet its effectiveness as a policy instrument against poverty remains unproven; we do not assess this causally here, and future research should test whether such discourse accelerates policy adoption within European countries. Child poverty warrants specific attention**,** as early-life deprivation has lasting effects on health, education, and earnings [74]; subsequent studies should therefore incorporate child-specific measures and subgroup analysis.

In conclusion, the findings highlight substantial and persistent heterogeneity in poverty realities within the EU, which cannot be fully understood without a multidimensional perspective. The novelty of the paper is the development of a dynamic, multi-period cluster typology that combines indicators of poverty and material deprivation with housing conditions and digital readiness, thereby making it possible to distinguish between stable and transitional forms of disadvantage. Consequently, the policy responses cannot be grounded solely in income indicator, but need to reflect also housing affordability, employability, education, and digital inclusion. At the same time, it is necessary to expand the range of measures to include innovative approaches, such as support for social entrepreneurship [75], investment in digital skills development, and links to the green transition, to foster sustainable development and resilience in the event of future crises. While the data reveal differences between clusters, including their strengths and weaknesses, differentiation should apply at the cluster level and, crucially, policy design should also recognise country-level specificities, reflecting intra-cluster heterogeneity and shifts of countries across cluster boundaries. Further research is required to confirm these patterns longitudinally and to compare the effectiveness of cluster-tailored and country-specific interventions.

## Supporting information

S1 File Contains S1-S12 Tables and S1-S6 Figs. (DOCX)
