# Supplementary material for: Multivariate analysis of poverty and social exclusion across EU countries in the context of Sustainable Development Goal 1
Source: PLoS One. 2026 Sep 30;21(9):e0343050. doi: 10.1371/journal.pone.0343050 (PMC13626335; doi:10.1371/journal.pone.0343050)
Supplement: Supplementary file 1 [file pone.0343050.s001.docx]

**SUPPORTING INFORMATION**

**S1 Table** Detailed description of indicators used

| **Indicator** | **Description** |
| --- | --- |
| ***x*_1_** | **Persons at risk of monetary poverty after social transfer**  People at risk-of-poverty are persons with an equivalised disposable income below the risk-of-poverty threshold, which is set at 60 % of the national median equivalised disposable income (after social transfers). The indicator is part of the multidimensional poverty index. |
| ***x*_2_** | **Severe material and social deprivation rate**  Severely materially or socially deprived persons have living conditions severely constrained by a lack of resources, they experience at least 7 out of 13 following deprivations items: cannot afford i) to pay rent or utility bills, ii) keep home adequately warm, iii) face unexpected expenses, iv) eat meat, fish or a protein equivalent every second day, v) a week holiday away from home, vi) have access to a car/van for personal use; vii) replace worn out furniture; viii) replace worn-out clothes with some new ones; ix) have two pairs of properly fitting shoes; x) spend a small amount of money each week on him/herself (“pocket money”); xi) have regular leisure activities; xii) get together with friends/family for a drink/meal at least once a month; and xiii) have an internet connection. The indicator is based on the EU-SILC (statistics on income, social inclusion and living conditions). |
| ***x*_3_** | **Persons living in households with very low work intensity**  People in the age of 0 to 64 years living in households where the adults worked a working time equal or less than 20 % of their total combined work-time potential during the previous year.  As adults count people in the age of 18 to 64 years. Students aged 18 to 24 years; people who are retired according to their self-defined current economic status or who receive any pension (except survivor’s pension); and people in the age bracket 60 to 64 who are inactive and living in a household where the main income is pensions, are not taken into account. |
| ***x*_4_** | **In work at-risk-of-poverty rate**  The indicator measures the share of persons who are employed and have an equivalised disposable income below the risk-of-poverty threshold, which is set at 60 % of the national median equivalised disposable income (after social transfers). For the purpose of this indicator, an individual is considered as being employed if he/she was employed for more than half of the reference year. The indicator is based on the EU-SILC (statistics on income, social inclusion and living conditions). |
| ***x*_5_** | **Housing cost overburden rate**  The indicator measures the share of population living in households that spend 40 % or more of the household disposable income on housing ('net' of housing allowances). Housing costs include rental or mortgage interest payments but also the cost of utilities such as water, electricity, gas or heating. |
| ***x*_6_** | **Severe housing deprivation rate**  Severe housing deprivation rate is defined as the percentage of population living in the dwelling which is considered as overcrowded, while also exhibiting at least one of the housing deprivation measures.  Housing deprivation is a measure of poor amenities and is calculated by referring to those households with a leaking roof, no bath/shower and no indoor toilet, or a dwelling considered too dark. |
| ***x*_7_** | **GDP per capita in PPS**  The volume index of GDP per capita in Purchasing Power Standards (PPS) is expressed in relation to the European Union average set to equal 100. An index value above 100 indicates that a country's GDP per capita is higher than the EU average, while a value below 100 signifies that it is lower. This index is often used to compare the economic well-being of populations. However, GDP includes certain components and transactions that are arguably less relevant to measuring a household's current material well-being. PPS is the technical term used by Eurostat to denote a common currency that eliminates the differences in price levels between countries allowing meaningful comparisons. PPS values are derived by dividing a country's economic aggregates in national currency by the respective PPPs. Data are primarily intended for cross-country comparisons rather than for comparisons over time. |
| ***x*_8_** | **Real GDP growth rate**  Gross domestic product (GDP) is defined as the value of all goods and services produced less the value of any goods or services used in their creation. For measuring the growth rate of GDP in terms of volumes, the GDP at current prices are valued in the prices of the previous year and the thus computed volume changes are imposed on the level of a reference year; this is called a chain-linked series. Doing so, price movements will not inflate the growth rate. Expessed as percentage change on the previous period and as percentage change on the previous period per capita. |
| ***x*_9_** | **High-speed internet coverage**  The indicator measures the share of households with fixed very high capacity network (VHCN) connection. Very high capacity network means either an electronic communications network that consists entirely of optical fibre elements at least up to the distribution point at the serving location, or an electronic communications network capable of delivering, under usual peak-time conditions, similar network performance in terms of available downlink and uplink bandwidth, resilience, error-related parameters, and latency and its variation.  Target: Digital Decade target of this indicator in the EU by 2030 is set to 100 % coverage |
| ***x*_10_** | **Share of individuals having at least basic digital skills**  This indicator measures the share of people aged 16 to 74 who have at least basic digital skills. It is a composite indicator based on selected activities performed by individuals on the internet in specific areas: information and data literacy, communication and collaboration, digital content creation, safety and problem solving. The indicator assesses digital skills classified into six levels, of which the two highest constitute the basic or above basic level of digital skills. The indicator is based on data from the EU survey on the use of ICT in households and by individuals.  Target: Digital Decade target of this indicator in the EU by 2030 is set to is 80 % |

Source: Eurostat (n.d.)

**S2 Table** Country codes

| Belgium | (BE) | Greece | (EL) | Lithuania | (LT) | Portugal | (PT) |
| --- | --- | --- | --- | --- | --- | --- | --- |
| Bulgaria | (BG) | Spain | (ES) | Luxembourg | (LU) | Romania | (RO) |
| Czechia | (CZ) | France | (FR) | Hungary | (HU) | Slovenia | (SI) |
| Denmark | (DK) | Croatia | (HR) | Malta | (MT) | Slovakia | (SK) |
| Germany | (DE) | Italy | (IT) | Netherlands | (NL) | Finland | (FI) |
| Estonia | (EE) | Cyprus | (CY) | Austria | (AT) | Sweden | (SE) |
| Ireland | (IE) | Latvia | (LV) | Poland | (PL) |  |  |

Source: Eurostat (<https://ec.europa.eu/eurostat/statistics-explained/index.php?title=Glossary:Country_codes>)

**S3 Table** Summary statistics of the indicators *x*_1_ – *x*_10_ across three time spans

Source: Eurostat, own calculations

**S4 Table** Pearson correlation coefficients of examined indicators in the first-time span

Source: Eurostat, own calculations

**S5 Table** Kaiser´s Measure of sampling adequacy, communalities and component loadings in the first-time span

Source: Eurostat, own calculations

**S6 Table** Pearson correlation coefficients of indicators in the second-time span

Source: Eurostat, own calculations

**S7 Table** Kaiser´s Measure of sampling adequacy, communalities and component loadings in the second-time span

Source: Eurostat, own calculations

**S8 Table** Pearson correlation coefficients of indicators in the third-time span

Source: Eurostat, own calculations

**S9 Table** Kaiser´s Measure of sampling adequacy, communalities and component loadings in the third-time span

Source: Eurostat, own calculations

**S10 Table** Hierarchical clustering model summary – first-time span, years 2015 - 2019

| *Model Summary: Hierarchical Clustering* | | | | | |
| --- | --- | --- | --- | --- | --- |
| Clusters | N | R² | AIC | BIC | Silhouette |
| 6 | 27 | 0.8651 | 31.02 | 46.57 | 0.3400 |

Source: Eurostat, own calculations

**S1 Fig.** Dendrogram tree of the cluster analysis (first-time span, years 2015 - 2019)

Source: Eurostat, own calculations

**S2 Fig.** Diagram of the clusters of EU countries in the first-time span (years 2015 - 2019)

Source: Eurostat, own calculations

**S11 Table** Hierarchical clustering model summary – second-time span, year 2020

| *Model Summary: Hierarchical Clustering* | | | | | |
| --- | --- | --- | --- | --- | --- |
| Clusters | N | R² | AIC | BIC | Silhouette |
| 5 | 27 | 0.8169 | 29.52 | 42.48 | 0.3300 |
| *Note.*  The model is optimized with respect to the *silhouette* value. | | | | | |

Source: Eurostat, own calculations

**S3 Fig.** Dendrogram tree of the cluster analysis (second-time span, year 2020)

Source: Eurostat, own calculations

**S4 Fig.** Diagram of the clusters of EU countries in the second-time span (year 2020)

Source: Eurostat, own calculations

**S12 Table** Hierarchical clustering model summary – third-time span, years 2021 – 2023

| *Model Summary: Hierarchical Clustering* | | | | | |
| --- | --- | --- | --- | --- | --- |
| Clusters | N | R² | AIC | BIC | Silhouette |
| 7 | 27 | 0.9044 | 32.97 | 51.11 | 0.4000 |

Source: Eurostat, own calculations

**S5 Fig.** Dendrogram tree of the cluster analysis (third-time span, years 2021 – 2023)

Source: Eurostat, own calculations

**S6 Fig.** Diagram of the clusters of EU countries in the third-time span (years 2021 – 2023)

Source: Eurostat, own calculations
